# Epigenetic dynamics of monocyte-to-macrophage differentiation

**DOI:** 10.1186/s13072-016-0079-z

**Published:** 2016-07-29

**Authors:** Stefan Wallner, Christopher Schröder, Elsa Leitão, Tea Berulava, Claudia Haak, Daniela Beißer, Sven Rahmann, Andreas S. Richter, Thomas Manke, Ulrike Bönisch, Laura Arrigoni, Sebastian Fröhler, Filippos Klironomos, Wei Chen, Nikolaus Rajewsky, Fabian Müller, Peter Ebert, Thomas Lengauer, Matthias Barann, Philip Rosenstiel, Gilles Gasparoni, Karl Nordström, Jörn Walter, Benedikt Brors, Gideon Zipprich, Bärbel Felder, Ludger Klein-Hitpass, Corinna Attenberger, Gerd Schmitz, Bernhard Horsthemke

**Affiliations:** 1 Institute for Clinical Chemistry and Laboratory Medicine, University Hospital Regensburg, Regensburg, Germany; 2 Genome Informatics, Institute of Human Genetics, University Duisburg-Essen, Essen, Germany; 3 Institute of Human Genetics, University Hospital Essen, University Duisburg-Essen, Hufelandstraße 55, 45147, Essen, Germany; 4 Bioinformatics and Deep Sequencing Unit, Max Planck Institute of Immunobiology and Epigenetics, Freiburg, Germany; 5 Max Delbrück-Center for Molecular Medicine, Berlin, Germany; 6 Max Planck Institute for Informatics, Saarbrücken, Germany; 7 Institute for Clinical Molecular Biology, Christian-Albrechts-University, Kiel, Germany; 8 Institute of Genetics/Epigenetics, Saarland University, Saarbrücken, Germany; 9 Deutsches Krebsforschungszentrum, Heidelberg, Germany; 10 Biochip Lab, Institute of Cell Biology, University Duisburg-Essen, Essen, Germany; 11 Caritas Krankenhaus St. Josef, Regensburg, Germany

**Keywords:** Monocyte, Macrophage, Epigenetics, Methylation, Enhancer, Next-generation sequencing, Ten eleven translocation methylcytosine dioxygenase, TET, DEEP, IHEC

## Abstract

**Background:**

Monocyte-to-macrophage differentiation involves major biochemical and structural changes. In order to elucidate the role of gene regulatory changes during this process, we used high-throughput sequencing to analyze the complete transcriptome and epigenome of human monocytes that were differentiated in vitro by addition of colony-stimulating factor 1 in serum-free medium.

**Results:**

Numerous mRNAs and miRNAs were significantly up- or down-regulated. More than 100 discrete DNA regions, most often far away from transcription start sites, were rapidly demethylated by the ten eleven translocation enzymes, became nucleosome-free and gained histone marks indicative of active enhancers. These regions were unique for macrophages and associated with genes involved in the regulation of the actin cytoskeleton, phagocytosis and innate immune response.

**Conclusions:**

In summary, we have discovered a phagocytic gene network that is repressed by DNA methylation in monocytes and rapidly de-repressed after the onset of macrophage differentiation.

**Electronic supplementary material:**

The online version of this article (doi:10.1186/s13072-016-0079-z) contains supplementary material, which is available to authorized users.

## Background

The differentiation of monocytes to macrophages is dependent on macrophage colony-stimulating factor (CSF1/MCSF) and modulated by inflammatory stimuli such as LPS, γ-IFN or TNFα. CSF1 promotes a resident-type macrophage phenotype with a role in tissue repair [1]. CSF1 binds to the extracellular domain of the CSF1 receptor (CSF1R) with downstream signaling via PI3K and MEK, to modulate differentiation and survival. Although much progress has been made in the understanding of macrophage activation, polarization and function, the underlying processes are still not fully understood.

A large transcriptomic data set of phagocyte differentiation and activation [1], among numerous other cells and tissues, has recently been released by the FANTOM consortium [2, 3]. All phagocytes express a small number of lineage-specific transcription factors (TFs) and an array of known lineage-specific genes [4]. Transcriptional changes are mainly mediated by the selection and establishment of enhancers (for review, see [5]). Based on mouse studies, it has been proposed that PU.1 and serum response factor (SRF) regulate cytoskeletal gene expression in macrophages [6]. Furthermore, miRNA signatures were identified in polarized macrophages that are differentially regulated during monocyte-to-macrophage differentiation and polarization [7].

The BLUEPRINT consortium recently reported some epigenetic aspects of monocyte-to-macrophage differentiation [8]. Monocytes from peripheral blood were enriched by antibody-based depletion of T, B and NK cells and differentiated in medium enriched with 10 % human serum as a source of CSF1/MCSF. Based on the analysis of three histone marks (H3K4me1, H3K4me3 and H3K27ac) as well as DNase I accessibility, the authors identified approximately 8000 dynamic regions and found that naïve macrophages displayed a remodeled metabolic enzyme repertoire and attenuated innate inflammatory pathways. DNA methylation was not analyzed in this study.

Changes in DNA methylation have previously been shown to occur in hematopoietic stem cells and during later stages of hematopoiesis. Using methyl-CpG immunoprecipitation and promoter microarrays, Klug et al. [9] observed active DNA demethylation during monocyte to dendritic cell (DC) differentiation, which was not necessarily linked to transcription changes. This group also showed that an siRNA-mediated knockdown of TET2 in primary monocytes prevented active DNA demethylation [10].

For a comprehensive analysis of ​monocyte-to-macrophage differentiation, we have generated class I reference epigenomes of both cell types according to IHEC standards. To avoid the confounding effects of antibody-based isolation techniques and serum-containing culture media, monocytes were purified by counterflow elutriation and differentiated in serum-free medium. By focusing on differential DNA methylation, we discovered a gene network regulating the actin cytoskeleton and phagocytosis.

## Results

For generating epigenomes, RNA, DNA and chromatin from the same batch of cells from two healthy male donors (Hm03 and Hm05) were used.

### Gene expression analysis

**Fig. 1:**
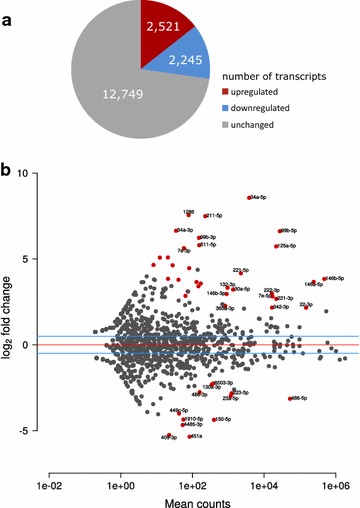
Transcriptional analysis. **a** Regulation of mRNA transcripts during ​monocyte-to-macrophage differentiation. **b** log2 transformed expression changes upon ​monocyte-to-macrophage differentiation plotted against miRNA levels in mean read counts. Significantly regulated miRNAs (p_adj_ < 0.05) are labeled and depicted in* red*

Transcriptome-wide analysis of mRNAs from monocytes and macrophages was performed by RNAseq, and gene expression differences were determined with the DESeq software package. Of the 17,515 expressed genes in monocytes and macrophages, 2521 genes were significantly up-regulated and 2245 genes were significantly down-regulated upon differentiation (padj <0.05, Fig. 1a). In order to investigate how our results compare to the BLUEPRINT data [8], where the authors used antibodies for cell isolation and serum-containing media, we correlated the expression differences in our cell system with that of their cell system. The Pearson product–moment correlation coefficient of the comparison was 0.68, indicating a moderate to strong correlation (Additional file 1: Figure S1).

Next, we had a look at major differences between the two data sets. By performing a gene ontology (GO) enrichment analysis of genes that were unchanged in our experiment, but up-regulated in the BLUEPRINT experiment, we found an overrepresentation of 12 terms, all of which are related to metabolic processes (Additional file 2: Table S1), suggesting that the “remodeled metabolic enzyme repertoire” described by Saeed et al. [8] might—at least in part—be related to the use of serum in the culture medium. The reverse analysis (genes unchanged in BLUEPRINT, but up-regulated in our experiment) revealed no significant enrichment of GO terms.

We also investigated whether ​monocyte-to-macrophage differentiation involves changes in miRNAs. As shown in Fig. 1b and in more detail in Additional file 3: Table S2, 34 miRNAs are significantly up-regulated and 12 miRNAs are significantly down-regulated after differentiation. Interestingly, several of the highly expressed and up-regulated miRNAs are encoded by miRNA gene clusters (miR-99b-5p, let-7e and miR-125a-5p on 19q; miR-222-3p and miR-221-3p on Xp; miR-23a-3p and miR-27a-3p on 19p).

We selected miRNAs that were found significantly more than twofold up- or down-regulated (Benjamini & Hochberg adjusted p value <0.05) and searched in the TargetScanHuman database (Release 7.1) for their family’s targets, demanding larger than 80 % probability of conserved targeting (TargetScan PCT score >0.8). In the whole list of 17,515 expressed genes in monocytes and macrophages, we found 3844 and 307 confident targets for the families of the up- and down-regulated select miRNAs, respectively. Interestingly, all of the 307 targets of the down-regulated miRNAs belonged to the group of 3844 targets of the up-regulated miRNAs as well, suggesting that those 307 targets are under constant miRNA interaction. A GO enrichment analysis found those 307 targets enriched in biological processes such as miRNA loading to RISC, signal transduction and cell differentiation among others (Benjamini & Hochberg adjusted p value <0.05).

We found an enrichment in the significantly differentially expressed genes among the confident targets of the selected miRNAs (Fisher’s exact test p value <1e−16). Furthermore, confident targets of only the up-regulated selected miRNAs were found enriched in both the significantly down-regulated genes (Fisher’s exact test p value <1e−16) and up-regulated genes, albeit with a weaker p value (Fisher’s exact test p value = 0.0002). However, the confident targets of only the up-regulated selected miRNAs were not enriched in the top 10 % of highest expressed and significantly up-regulated genes (Fisher’s exact test p value = 0.7). Taken together, these results suggest that a major part of miRNA functionality is dedicated to target suppression and another part involves target thresholding that is maintained throughout the differentiation transition. These conclusions are in perfect agreement with previous work based on a tissue-wide study of miRNA expression [11], where it was found that predicted targets are expressed at lower levels when co-expressed with their targeting miRNAs, whereas highly expressed genes tend not to be targets of co-expressing miRNAs.

### DNA methylation analysis

**Fig. 2:**
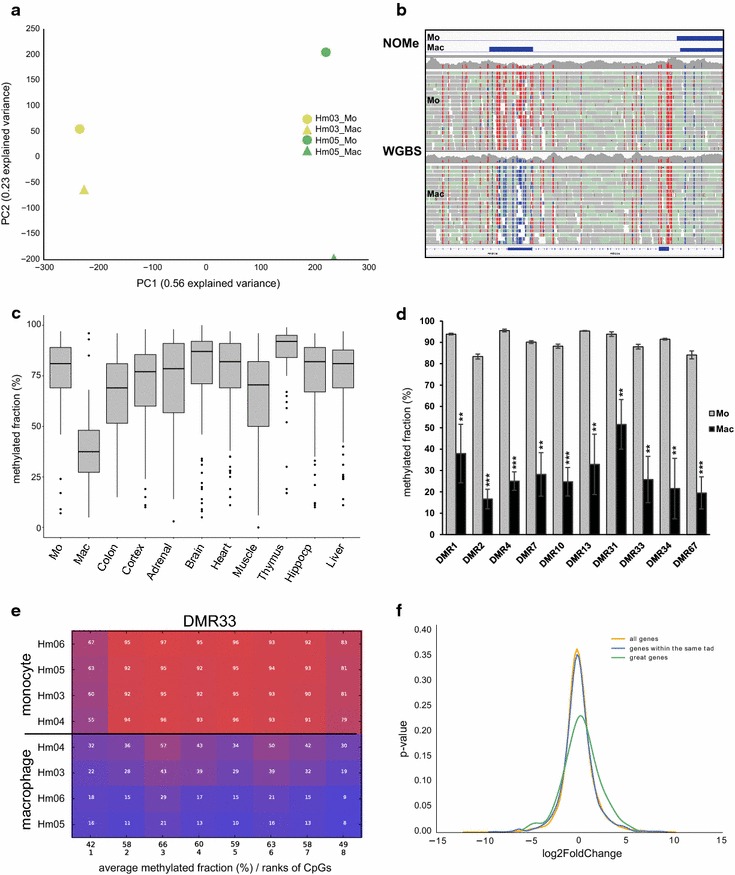
DNA methylation analysis. **a** PCA on whole-genome bisulfite data. A clear separation of monocytes (Mo) and macrophages (Mac) as well as donors Hm03 and Hm05 is visible. **b** Representative example of a DMR in the left part of an IGV browser snapshot. The DMR coincides with the gain of a NOMe peak. **c** Comparison of the average methylated DNA fraction in the identified DMRs in a variety of tissues subjected to WGBS [12].* Boxplots* showing medians, 75th and 25th percentile (*box limits*) and whiskers of 1.5 × IQR, p < 0.0001 (unpaired Student’s t test) for Mac against all other tissues. **d** Validation of 10 DMRs by targeted deep bisulfite sequencing. Average CpG methylated fractions in monocytes and macrophages after at least 3 days of differentiation. Results are mean ± SD from 4 independent donor samples (Hm03, Hm04, Hm05 and Hm06). **p value <0.01; ***p value <0.001 (two-tailed paired Student’s t test). Related to Additional file 6: Figure S3. **e** Representative comparative methylation plot (DMR33). Samples are sorted by overall methylation. Related to Additional file 6: Figure S3. **f** Distribution plot of expression changes for all genes (*yellow*), genes within the same TAD (*blue*) and genes identified in the GREAT analysis (*green*)

For analyzing DNA methylation at single base-pair resolution, we did whole-genome bisulfite sequencing (WGBS). By performing a principal component analysis on the WGBS data set, we found that principal component 1 (PC1) separates the donors and principal component 2 (PC2) separates monocytes and macrophages (Fig. 2a). The genetic basis of the inter-individual differences in DNA methylation will be reported in more detail in a separate study.

### Gene expression changes are not correlated with DNA methylation changes at transcription start sites

In order to determine whether the gene expression changes occurring during ​monocyte-to-macrophage differentiation are related to DNA methylation changes around the transcription start sites of the genes, we performed a correlation analysis. As shown by the scatter plot (Additional file 4: Figure S2, Pearson’s r = −0.00018, p value = 0.97), there is no significant correlation. This finding raised the question whether DNA methylation changes occur in other regions of the genome and what function they might have.

### Identification of differentially methylated regions (DMRs)

For identifying differentially methylated regions (DMRs) between the two cell types, we performed an unbiased search using the software package BSmooth. Since WGBS studies are heavily underpowered with regard to the detection of methylation differences at single CpGs, we looked at strings of four or more CpGs. By setting the minimal mean methylation difference to 0.5, 0.4, 0.3, 0.2 and 0.1, we observed 1, 18, 114, 288 and 468 DMRs, respectively. With the aim of focusing on large methylation differences, we continued our study with the 114 DMRs that have a methylation difference equal or greater than 0.3 (Additional file 5: Table S3). The size of these DMRs varies between 85 and 1697 bp. While only 4/114 DMRs showed an increase in methylation, 110/114 DMRs were demethylated during ​monocyte-to-macrophage differentiation (one example shown in Fig. 2b). In published data from other somatic tissues also subjected to WGBS, most of these DMRs are highly methylated (p < 0.0001, unpaired Student’s t test; Fig. 2c) [12, 13].

Of all 114 DMRs, 15 were selected for validation by targeted deep bisulfite sequencing in cells from the donors used for WGBS (Hm03 and Hm05) as well as from two additional donors (Hm04 and Hm06). Priority was given to DMRs with >10 CpGs and experimentally verified transcription factor binding sites that are subject to chromatin opening (see below) during ​monocyte-to-macrophage differentiation (Fig. 2b). For 10 DMRs, we could establish reliable targeted bisulfite assays. In each case, the macrophages show CpG demethylation as compared to monocytes (Fig. 2d, e, related to Additional file 6: Figure S3), thus confirming the results obtained by WGBS.

### Demethylation of DMRs is linked to a macrophage-specific regulatory gene network

Since DNA demethylation is known to coincide with transcriptional activation, we analyzed these regions in further depth. Only two of the 114 regions overlap a CpG island as defined by Gardiner-Garden and Frommer [14]. This observation is in line with our finding that there is no correlation between gene expression changes and DNA methylation changes around the transcription start sites of gene (Additional file 4: Figure S2). Interestingly, 11/114 DMRs contain an exon, but there was no evidence for alternative splicing (data not shown).

To predict the function of the DMRs, we used the Genomic Regions Enrichment of Annotations (GREAT) tool [15], which assigns biological meaning to a set of noncoding genomic regions by analyzing the annotations of the nearby genes (for details, see “Methods”). We found 11 regions that are associated with one gene and 103 regions that are associated with two genes. Interestingly, most of the DMRs map far from the transcription start site of its associated gene (Additional file 7: Figure S4). Gene ontology analysis of the associated 217 genes against the whole-genome background revealed significant enrichment of 16 partially redundant GO terms, which include Fcγ receptor signaling, phagocytosis, cellular component organization, response to growth factor stimulus, immune response and regulation of metabolic processes. Ninety-five of the 217 DMR-associated genes belong to one or more of these terms (Additional file 8: Table S4).

**Fig. 3:**
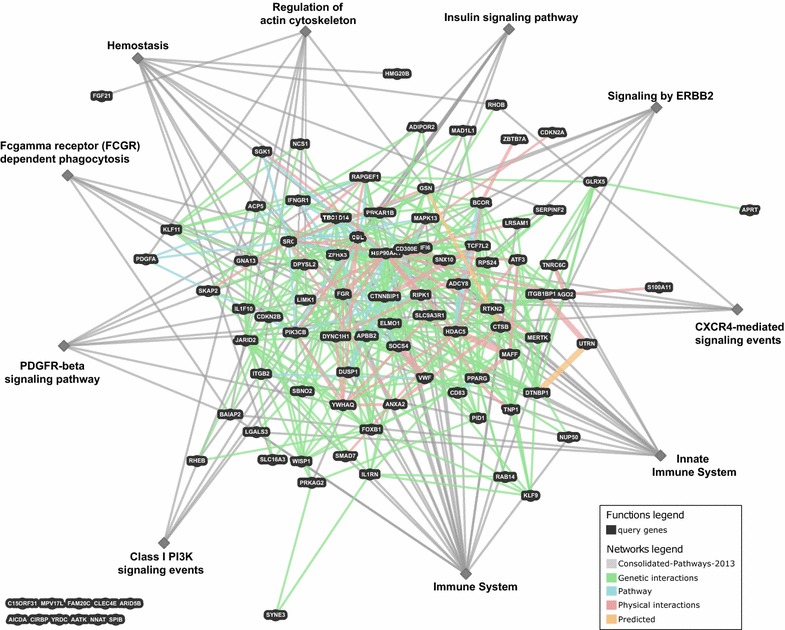
Network showing genetic, physical, pathway and predicted interactions between the 95 DMR-associated genes that belong to one or more of the 16 significantly enriched GO terms. For a more detailed inspection, the 95 genes (Additional file 8: Table S4) can be uploaded to http://www.genemania.org/

In order to determine whether demethylation of the DMRs is associated with expression changes of nearby genes, we first compared the distribution of expression changes of all genes sharing a topological domain (TAD, [16]) with a DMR (n = 1160) to the distribution of expression changes of all expressed genes (n = 17,515). As shown in Fig. 2f, no difference was observed (yellow and blue curves; Kolmogorov–Smirnov test, p = 0.47). Next, we analyzed those 95 DMR-associated GO genes identified above (green curve). In this case, the distribution of expression changes was significantly different to that of all genes (Kolmogorov–Smirnov test, p = 0.028), suggesting that the DMRs participate in control of the expression of a significant fraction of these genes. Most of the 95 genes form an interaction network as shown in Fig. 3. Forty-three of these genes are part of ten consolidated pathways related to the regulation of actin cytoskeleton, phagocytosis and innate immune system.

### DMRs are enriched for specific transcription factor binding motifs

Almost all of the DMRs (103/114) contain a transcription factor binding site as identified by chromatin immunoprecipitation (ENCODE Txn Factor ChIP data, Additional file 5: Table S3). To test for significance, we simulated 1 million sets, each consisting of 114 regions of equal length compared to the original DMRs and each region containing at least 4 CpGs. Only 21 simulations showed an equal or higher fraction of transcription factor binding sites (empirical p value = 0.000021).

We also asked which transcription factor binding motifs are enriched in the DMRs. To this end, we used the TRANSFAC database (professional version, release 2015.3; http://www.biobase.de/product/transcription-factor-binding sites) [17]. Random regions and provided example regions served as background, and the parameters were set to default. Depending on the data set used as a background, we found that 13 (experimental data) and 19 (randomly generated data) transcription factor binding motifs were significantly enriched (p < 0.01) in the DMRs (Table 1). Eight motifs were found in both comparisons. Some of these transcription factors are known to play a role in phagocytic differentiation (GKLF/KLF4 and SREBP, [18–20]) and/or to be transcription factors that can open closed chromatin (AP-1, RFX1, GKLF/KLF4 and p65 [21]).

**Table 1:** Lists of overrepresented transcription factor binding motifs in differentially methylated regions using the Transfac database and default settings for the experimental and the random data set

Factor	Versus experimental set (A)			Versus random set (B)		
	Yes	No	Yes/no	Yes	No	Yes/no
AP-1	0.1667	0.0038	43.6333	0.1667	0.0175	9.5000
RelA-p65	0.1404	0.0130	10.8070	0.1140	0.0175	6.5000
MAF	0.1754	0.0260	6.7544	0.1140	0.0088	13.0000
Muscle initiator	0.1228	0.0191	6.4302	0.1667	0.0439	3.8000
GEN_INI	0.4474	0.0718	6.2298	0.1053	0.0088	12.0000
GKLF	0.6140	0.1406	4.3683	0.6140	0.0877	7.0000
Myogenin	0.9474	0.2231	4.2469	0.9474	0.2632	3.6000
CPBP	2.0877	0.6157	3.3906	2.0877	1.0877	1.9194
BBX secondary motif	0.0439	0.0023	19.1374			
RFX1	0.0526	0.0038	13.7789			
TTF-1	0.1404	0.0237	5.9264			
AHR	0.2281	0.0413	5.5286			
ZNF333	0.8772	0.5225	1.6787			
Ikaros				0.5965	0.0263	22.6667
SREBP				0.1404	0.0088	16.0000
Ets				0.1140	0.0088	13.0000
Helios A				0.1140	0.0088	13.0000
MZF-1				0.2193	0.0526	4.1667
Smad4				0.1842	0.0526	3.5000
NF-AT1				0.5351	0.1754	3.0500
MAFA				0.2105	0.0702	3.0000
FAC1				0.2456	0.0877	2.8000
ING4				0.6316	0.2368	2.6667
SRY				0.6579	0.2895	2.2727

Yes and No denote the relative number of sites for the selected matrix in the DMRs as compared to an experimental data set (A) and a random data set (B)

### Most of the DMRs become nucleosome-depleted

In order to investigate whether the chromatin accessibility of the DMRs changes during differentiation, we performed NOMe-seq (**N**ucleosome **O**ccupancy and **Me**thylation assay) on cells from donor Hm05. In this technique, native chromatin is treated with the GpC-specific methyltransferase M.CviPI which methylates GpC dinucleotides in nucleosome-free regions [22]. The DNA is then treated with sodium bisulfite and subjected to WGBS. From these data, CpG methylation patterns as well as nucleosome-free regions (GpC methylation) can be identified. First, we used a NOMe-seq peak finder (Nordström et al., unpublished) to identify nucleosome-depleted regions in the two lineage-specific differentiation stages. While monocytes had 89,212 NOMe-seq peaks, covering about 21 Mbp, macrophages had 127,267 peaks covering about 42 Mbp which demonstrates that macrophages have more nucleosome-depleted regions. We then determined the overlap between DMRs and NOMe-seq peaks that were gained or lost during differentiation. While 21/114 DMRs did not overlap with a NOMe-seq peak in any cell type, 85/114 (74.6 %) DMRs gained a peak and 2/114 (1.8 %) lost a peak. In six cases, the NOMe-seq peak had different borders. We conclude that most DMRs become nucleosome-depleted during differentiation.

### DMRs have distinct histone modifications

**Fig. 4:**
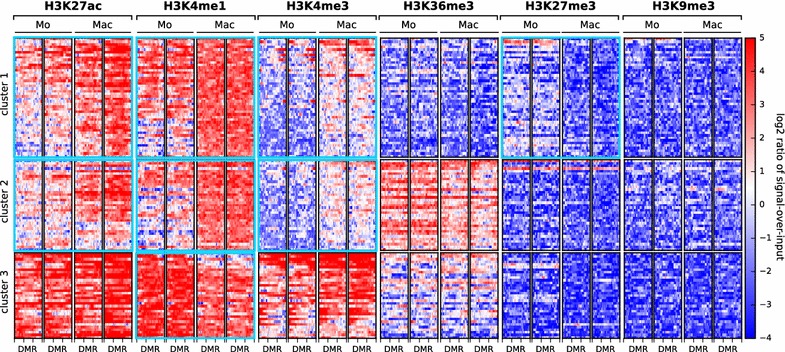
Heatmap of histone modification signals at 114 differentially methylated regions in monocyte (Mo) versus macrophage (Mac). The heatmap shows the log2 ratio of ChIP signal over input at the DMRs plus 1 kb flanking regions for six different histone modifications. k-means clustering revealed three cluster with distinct chromatin signatures. Cluster 1 comprises DMRs that gain in macrophages the signature of active enhancers in non-transcribed regions, cluster 2 comprises DMRs that gain in macrophages the signature of active enhancers in actively transcribed regions and cluster 3 comprises DMRs with the signature of promoters or enhancers that are active in both monocyte and macrophage. Clusters and histone marks with significant change during ​monocyte-to-macrophage differentiation (p < 0.001, paired Student’s t test with Bonferroni correction) are highlighted with a *bold light blue* border

To gain further insight into the function of the DMRs, we used ChIP-seq to profile the histone modifications H3K27ac, H3K4me1, H3K4me3, H3K36me3, H3K27me3 and H3K9me3. DMRs plus 1 kb flanking sequences were clustered by ChIP signal over input log2 ratio across all six histone marks using the *k*-means algorithm. Three clusters with distinct chromatin signatures were found (Fig. 4, [23]). The DMRs in clusters 1 and 2 showed a significantly increased signal of the histone marks H3K27ac and H3K4me1 in macrophages compared to monocytes (p < 0.001, paired Student’s t test with Bonferroni correction). The presence of these two marks is a characteristic of active enhancers. The regions in both clusters also gained H3K4me3 (p < 0.001, paired Student’s t test with Bonferroni correction), a mark found at promoters and certain enhancers, during ​monocyte-to-macrophage differentiation. The regions in cluster 1 additionally became reduced in the repressive mark H3K27me3 during differentiation (p < 0.001, paired Student’s t test with Bonferroni correction), which complements the gain of H3K27ac. Cluster 2, in contrast to cluster 1, comprises DMRs that are consistently decorated with H3K36me3, which associates with actively transcribed gene bodies. The DMRs in cluster 3 were marked with H3K27ac, H3K4me1 and H3K4me3 in both monocytes and macrophages. Although the H3K4me1 signal was significantly reduced in macrophages, the other marks were without significant change during differentiation (p < 0.001 for H3K4me1, p > 0.001 for the remaining marks; paired Student’s t test with Bonferroni correction). These results suggest that the DMRs in cluster 3 are located at promoters or enhancers that are active in both cell types.

As our consortium did not investigate enhancer RNAs (eRNAs), we queried the FANTOM database for bidirectional eRNAs in monocytes and macrophages. This revealed that 24 of the 114 identified DMRs are likely to produce bidirectional eRNAs indicating enhancer activity. In 21 of these cases, the FANTOM-predicted target DNA of the enhancer is the transcription start site of the DMR host gene.

### Demethylation is a rapid process and likely mediated by Tet dioxygenases

**Fig. 5:**
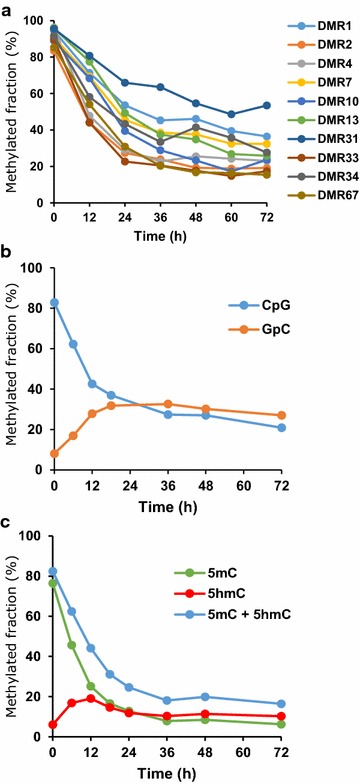
Time-course analysis. **a** Demethylation of 10 DMRs. Average CpG methylated fractions in Hm06 donor monocytes (0 h) and cells collected at different time points during differentiation into macrophages. Related to Additional file 9: Figure S5. **b** Time course of chromatin accessibility (GpC methylation in NOMe experiments) and DNA CpG methylation in DMR33. Average CpG and GpC methylated fractions in Hm10 donor monocytes (0 h) and cells collected at different time points during differentiation into macrophages. The transition from lower to higher GpC methylated fraction is indicative of an increase in chromatin accessibility. GCG motifs were excluded due to ambiguity between CpG- endogenous- and GpC-enzymatic-methylation. Related to Additional file 10: Figure S6. **c** 5-hydroxymethyl-cytosine (5hmC) level increases during differentiation. Average 5hmC and 5mC (5-methylcytosine) fractions in monocytes (0 h) and cells collected at different time points (DMR33 from donor Hm10). Related to Additional file 14: Figure S7

In order to determine how rapidly the identified regions become free of methylation and nucleosomes, we differentiated monocytes to macrophages (donor Hm06) and removed aliquots at several time points. By analyzing CpG levels in ten DMRs at each time point (Fig. 5a, related to Additional file 9: Figure S5), we observed a rapid decline in DNA methylation within the first 24 h. By a time-course analysis of DNA methylation (CpG) and chromatin accessibility (GpC in NOMe-seq) of five DMRs in donor Hm10, we observed that chromatin opening also occurs very rapidly (most pronounced at 6–18 h, Fig. 5b and Additional file 10: Figure S6). These data validate WGBS results and suggest that both the opening of these genomic regions and their DNA demethylation occur simultaneously in the early stages of the differentiation process.

Monocytes do not divide during differentiation, which makes passive loss of DNA methylation unlikely. Also, mRNA levels of the maintenance methyltransferase *DNMT1* as well as of *DNMT3A* and *DNMT3B* remained stable. Therefore, we anticipated active DNA demethylation by oxidation. To further study this process, we investigated the dynamics between 5-methylcytosine (5mC) and the first oxidative product of active DNA demethylation, 5-hydroxymethylcytosine (5hmC) in two DMRs (DMR13 and DMR33) in both donor Hm06 and donor Hm10. Again, we analyzed cell aliquots at multiple time points. We observed that the oxidation of 5mC was particularly fast in the first 12 h, with cells exhibiting methylation levels of approximately 20 % 5hmC after 12-h differentiation, corresponding to a threefold to 13-fold increase relative to monocytes (Fig. 5c and Additional file 10: Figure S6).

**Fig. 6:**
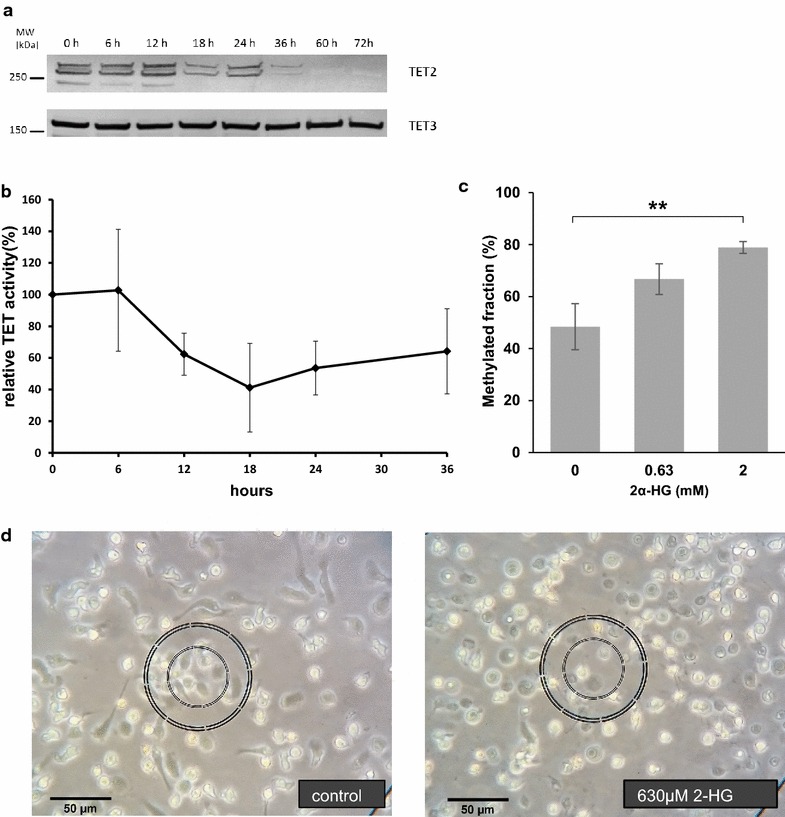
Analysis of TET activity. **a** Western blot for TET enzymes (donor Hm14) showing a decrease in TET2 protein levels during the first day of differentiation. Multiple isoforms are detectable for TET2. TET3 levels did not change. TET1 was undetectable by western blotting using commercial antibodies. **b** TET activity during ​monocyte-to-macrophage differentiation. TET activity in nuclear extract decreases significantly (p < 0.05, ANOVA) during differentiation from freshly isolated monocytes at 0 h to adherent phagocytic cells at later time points (n = 3–5, mean ± SEM). **c** Quantification of DMR33 methylation after 1-day differentiation exposed to different concentrations of the TET inhibitor Octyl-2-α hydroxyglutarate (2-HG). Results are mean ± SD from 3 independent donor samples (Hm15, HU2 and HU3). **p value <0.01 (unpaired Student’s t test). (D) Light microscopy of monocytes after 1 day differentiation in the presence or absence of TET inhibitor (donor Hm15). While control cells acquire a macrophage-like, adherent phenotype with protrusions, cells treated with 630 µM 2-HG are only loosely attached and have a round phenotype

Since conversion of 5mC into 5hmC is performed by members of the ten eleven translocation family (TET), we further investigated the involvement of these enzymes [12]. Monocytes show very low levels of *TET1* mRNA, while *TET2* and *TET3* are substantially expressed. *TET2* and *TET3* mRNA levels decrease during differentiation (*TET2*, log2FC = −2.07, adjusted p value = 1.32E−6; *TET3*, log2FC = −1.2, adjusted p value = 0.007). At the protein level, a substantial decrease in TET2 occurred in the first 24 h (Fig. 6a). Since we expected regulation to take place not only on the mRNA and protein level, we performed an in vitro TET hydroxylase assay to check whether TET hydroxylase activity is modulated during differentiation. As shown in Fig. 6b, TET activity dropped considerably during the first 12 h and subsequently reached a lower steady level.

To test whether TET-mediated demethylation is essential for ​monocyte-to-macrophage differentiation, we decided to inhibit the TET enzymes. In view of the fact that demethylation occurs very rapidly (see above), whereas an antisense-mediated TET knock-down would become effective only after 48 h, we decided to treat freshly isolated monocytes from three independent donors (Hm15, HU2 and HU3) with the cell-permeable, competitive dioxygenase inhibitor (2S)-Octyl-alpha-hydroxyglutarate (2-HG). 2-HG has been reported before to be able to inhibit TET activity [24]. With this approach, we were able to inhibit DNA demethylation of DMR33 partially (630 µM) or completely (2 mM) (Fig. 6c, compare also to Fig. 5) as compared to the methylation levels of monocytes (Fig. 2d). The inhibitor prevented the formation of cellular protrusions and adherence (Fig. 6d). In contrast, flow cytometry for early macrophage differentiation markers (CD14, CD71, CD81, CD11b, CD195, CD68 and CD16) after 1 day of differentiation did not show any difference between 2-HG treated and control cells (data not shown). Apoptosis as detected by staining with Annexin-V/7-AAD was increased in cells treated with the inhibitor (33 % in cells treated with 2 mM 2-HG vs 5 % for treatment with 630 µM 2-HG and 2 % in untreated control cells). Since monocyte DNA methylation levels were significantly maintained (~ 50 %) at 630 µM and completely maintained at 2 mM (see above), the vast majority of methylated DNA molecules cannot be derived from dead cells.

## Discussion

Based on high-throughput epigenome analysis according to IHEC standards, we found that ​monocyte-to-macrophage differentiation is characterized by extensive changes in mRNA and miRNA levels. These changes are reflected by widespread changes in histone modifications and chromatin accessibility ([8] and our study). In contrast, DNA methylation changes affect only a small number of genomic regions, and most of them are far away from transcription start sites. Notably, these regions are enriched for binding sites of transcription factors such as AP-1, RFX1 and KLF4, which can open chromatin, are rapidly demethylated by TET enzymes, become nucleosome-free and gain active enhancer marks. DNA demethylation affects a small and specific set of genes, which regulate the actin cytoskeleton and phagocytosis and thus are most important for macrophage structure and function. This network remains repressed by DNA methylation in other somatic tissues.

Several of the genes regulated by the DMRs are part of ERBB2, PDGFRβ, CXCR4 and PIK3 signaling pathways (Fig. 3), which are involved in regulating actin cytoskeleton and phagocytosis (for review, see, for example, [25]). ERBB2 stabilizes ligand binding and enhances kinase (PI3K)-mediated activation of downstream signaling pathways, regulates cytoskeletal arrangements and outgrowth and stabilization of peripheral microtubules involved in podia formation, cell migration and phagocytosis. PDGFRβ signaling promotes rearrangement of the actin cytoskeleton, membrane ruffling and podia formation. Furthermore, it is involved in chemotaxis and initiating intracellular signaling through the MAPK, PI3K and PKCγ gamma pathways. Engagement of Fcγ receptors by IgG-opsonized particles leads to their clustering and tyrosine phosphorylation by Src-family kinases. This event in turn recruits the tyrosine kinase Syk as well as phosphatidylinositol 3′-kinase (PI3K). The formation of pseudopods is coincident with local remodeling of the underlying actin cytoskeleton to form a dense heavy mesh termed the actin cup. Actin cup formation requires, among others, activation of Rho-family GTPases [25]. In addition, other genes such as *GSN* and *ELMO1* are related to the actin cytoskeleton and phagocytosis. Several other genes are important for the (innate) immune system (e.g., *IFI6* and *IL1RN*).

Although we have not found any DNA methylation changes at miRNA genes, it is interesting to note that at least three miRNAs that are highly expressed and strongly up-regulated during ​monocyte-to-macrophage differentiation (miR-34, miR-146 and miR-221) have been reported to play a role in actin cytoskeletal reorganization in macrophages or other cell types [26–28].

Many genes in the identified network do not change their level of expression during macrophage differentiation (Fig. 2f, green line, and Additional file 8: Table S4). In an independent DNA methylation study based on 450 K microarrays, it was found that demethylated genes become activated only after cell stimulation [29]. It is possible that RNA polymerase II is stalled at the promoter of these genes, poising them for a prompt and coordinated expression in response to future stimuli. In fact, this mechanism of controlling the timing and amplitude of transcriptional responses was reported to occur in macrophages for several pro-inflammatory genes that have PolII paused at their promoters and basal levels of transcription prior to stimulation [30, 31].

DNA demethylation during ​monocyte-to-macrophage differentiation is a rapid, active process catalyzed by TET enzymes, as shown by increasing levels of 5hmC levels in the first 12 h of differentiation and the inhibition of this process by the competitive dioxygenase inhibitor 2α-hydroxyglutarate. Our results are in line with data showing active demethylation and a role for TET2 in monocyte differentiation [9, 10, 29, 32]. Zhang et al. [32] showed that when differentiating to dendritic cells, TET2 was up-regulated on the first day and then showed decreasing levels. Also similar to our data, no regulation of TET3 was observable [32]. Furthermore, in dendritic cells half of DMR-associated genes were up-regulated during differentiation, and DMRs were not confined to proximal promoters [9]. Since TET activity is high in monocytes, it seems to act as a standby system that is provisioned in monocytes and rapidly targeted to specific genomic regions upon induction of differentiation. Although the mechanistic details are unknown, it is likely that the TET enzymes are recruited to the target regions by pioneer transcription factors or other factors following the pioneers. Currently, our data do not allow us to decide whether transcription factor binding follows TET-mediated opening of chromatin or whether pioneer transcription factors recruit TET proteins to the DMR. Potentially “both mechanisms operate and reinforce one another” [33].

A high concentration of the TET inhibitor 2-HG, which completely inhibited DNA demethylation, prevented the formation of cellular protrusions and adherence, whereas the appearance of early cell surface markers was not affected. This finding substantiates the notion that demethylation leads to derepression of a specific program involved in cell structural changes and phagocytosis. Cells treated with higher TET inhibitor concentrations showed increased apoptosis, which could be due to the inability of developing adherence. However, it should be noted that 2-HG also inhibits other 2-ketoglutarate-dependent dioxygenases, for example histone demethylases. Thus, the effect of TET inhibitor on the cellular morphology may also be due to changes in histone modifications patterns.

## Conclusions

**Fig. 7:**
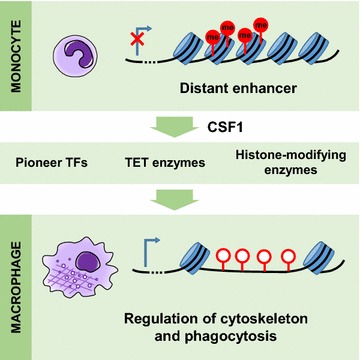
Graphical summary. A phagocytic gene network that is repressed by DNA methylation in monocytes is rapidly derepressed after the onset of macrophage differentiation

In summary, we have found that a specific gene network related to phagocyte structure and function is repressed in monocytes and other cells by DNA methylation of distant enhancers and that this program is rapidly derepressed by CSF1-induced demethylation of these regions (Fig. 7). Thus, ​monocyte-to-macrophage differentiation is a prime example for the role of targeted demethylation in cell differentiation.

## Methods

### Monocyte elutriation and differentiation

Primary human monocytes were obtained from healthy normolipidemic volunteers with the apolipoprotein E3/E3 genotype by leukapheresis and counterflow elutriation as described previously [34]. The study was approved by the ethics committee of the University Hospital Regensburg, and donors gave their written consent (Universitätsklinikum Regensburg, Ethikkommission der medizinischen Fakultät, proposal 08/119). Monocyte cultivation and differentiation were conducted according to Stöhr et al. [35]. Briefly, elutriated monocytes were seeded at 1 × 10^6^ cells/mL in macrophage serum-free medium (Invitrogen, Germany) supplemented with 50 ng/mL recombinant human monocyte colony-stimulating factor (rhMCSF; R&D Systems, USA) to induce phagocytic differentiation. Cells were cultured on plastic petri dishes (Sarstedt, USA) and incubated at 37 °C/5 % CO_2_ for five days. For time-course experiments, cells were collected at different time points during differentiation (0 h, 12 h, 24 h, 36 h, 48 h, 60 h and 72 h; 6 h and 18 h time points were additionally performed once). For ChIP-seq and NOMe-seq, cells were fixed for 5 min in 1 % paraformaldehyde (Sigma-Aldrich). Fixation was stopped using 125 mM glycine, and cells were thoroughly washed in PBS. Cell pellets were shock-frozen in liquid nitrogen and stored at −80 °C until analysis. Donors used for each type of experiment are summarized in Additional file 11: Table S5. Sample names were assigned according to the standardized DEEP naming scheme with, e.g., Hm03 representing cells from human, male donor 03. A more detailed overview can also be found in Additional file 11: Table S5.

### Magnetic bead-based isolation of monocytes

Bisulfite sequencing in the TET inhibitor experiments (donors Hm15, HU2 and HU3) required only a low number of cells. Therefore, monocytes for these assays were isolated by negative magnetic bead selection (Pan-monocyte isolation kit, #130-096-537, Miltenyi-Biotec) on an Auto-MACS system (Miltenyi Biotec) and in vitro differentiated as described above for elutriated cells.

### Nucleic acid isolation

Total RNA was isolated using PeqGOLD TriFast reagent (PeqLab, Germany) according to manufacturer’s recommendation. QIAamp columns (Qiagen, Germany) were used for DNA isolations. Nucleic acids were quantified with a Nanodrop 100 spectrophotometer (Peqlab, Germany) and RNA quality checked with an Agilent 2100 bioanalyzer on RNA 6000 nanochips.

### mRNA analysis

Long RNA libraries were prepared from 500 ng total RNA input of RNA integrity (RIN) >8 according to the Illumina stranded total RNA and mRNA protocols, respectively. In brief, poly-A enrichment (mRNA-seq) or ribosome-depletion (totalRNA-seq) was performed on the input total RNA, respectively. First- and second-strand cDNA synthesis and chemical fragmentation were performed, followed by adapter ligation and PCR amplification of the final library. PCR cleanup was performed using AMPure XP beads. Barcoded long RNA libraries were sequenced for 2 × 101 nt on an Illumina HiSeq 2000. Raw reads were aligned with TopHat2 [36] to the human reference genome (hs37d5). DESeq2 [37] with default settings was used to analyze differential gene expression between monocytes and macrophages using GENCODE reference annotation.

### miRNA analysis

Small RNA libraries were prepared from 1 µg total RNA of RIN >8 according to the Illumina small RNA protocol. In brief, adapter ligation, reverse transcription and PCR amplification were performed. Size selection on the library was performed by gel excision to capture the ~ 148nt fragment to contain the adapter-ligated small RNAs. Small RNA libraries were sequenced for 1 × 51 nt on an Illumina HiSeq 2000.

The expression levels of precursor hairpins and mature miRNAs obtained from miRBase (version 21) were estimated using the miRDeep2 algorithm (version 2.0.0.7) [38]. The total count of each mature miRNA and for each library was estimated by summing over the counts of identical mature miRNAs originating from different precursor hairpins. Differential expression analysis was done using the DESeq2 package [37].

### DNA methylation analysis

For each sample, two libraries were generated. A pre-bisulfite library was prepared from 2 µg of DNA according to the Illumina protocol essentially as described by Rademacher et al. [39]. A post-bisulfite library was prepared from 1 µg of DNA using the EpiGnome Methyl-Seq Kit from Epicentre. For each methylome, we sequenced two to three lanes of the Illumina library and one lane of the EpiGnome library on an Illumina HiSeq 2500, again as described previously [39].

The read mapping of the 650–700 million read pairs per sample was executed by bwa [40] version 0.7.5a and a modified human reference (hs37d5) to achieve C/T-tolerance. The segmentation into LMRs and UMRs was performed by MethylSeekR [41] version 1.6.0 with a FDR cutoff of 0.05. An in-house script was used to call the CpG methylation levels, excluding reads with a mapping quality lower than 30 (probability to map at a correct position = 0.999) and ignoring bases with base quality <17 (probability that the sequencing process produced the correct base = 0.98). BSmooth [42] was applied to detect DMRs with a minimum difference in methylation level of 0.3, a minimum size of four CpG and a q value cutoff of 0.0001 per single CpG. No region-wise p value or FDR is available for calculations with this tool.

All other statistical analyses and DMR annotations were performed by in-house scripts using R, SciPy and FANTOM [2] data of monocytes and macrophages for active enhancer, RefSeq [43] genes and CpG islands [16].

### NOMe-seq

One million fixed frozen cells were thawed in nuclei extraction buffer (60 mM KCl; 15 mM Tris–HCl, pH 8.0; 15 mM NaCl; 1 mM EDTA, pH 8.0; 0.5 mM EGTA, pH 8.0; 0.5 mM spermidine free base) supplemented with complete protease inhibitor cocktail (Roche, Basel, Switzerland) and 0.1 % NP40 (Sigma-Aldrich, St. Louis, USA), and incubated on ice for 30 min. During incubation, each sample was dounced 10–20 times with a douncing pistil (Qiagen, Hilden, Germany) until nuclei became visible under a standard light microscope. Nuclei were centrifuged (500*g*, 4 °C, 8 min), and the pellet was washed using the same buffer without NP-40. After another round of centrifugation, the pellet was gently resuspended in 90 µl of 1× GpC-buffer (NEB, Ipswich, USA) followed by addition of 70 µl of NOMe reaction mix 7 µl 10× GpC buffer (NEB), 1.5 µl of 32 mM SAM (NEB), 45 µl of 1 M sucrose, 60 U of M. CviPI (NEB) and 1 µl of lambda DNA spike-in (1 ng/µl). The reaction was incubated 3 h at 37 °C, and 0.5 µl of SAM was added after one and two hours. The reaction was stopped by adding 160 µl NOMe stop buffer (20 mM Tris–HCl, pH 8.0; 600 mM NaCl; 1 % SDS, 10 mM EDTA) and 10 µl proteinase K (20 mg/ml, Sigma-Aldrich), and genomic DNA was extracted. Next, 100 ng was bisulfite-converted with the EZ DNA Methylation-Gold kit (Zymo, Irvine, USA) and then subjected to NGS library preparation using the TruSeq DNA Methylation Kit (Illumina, San Diego, USA) according to the manufacturer’s protocol. All libraries were checked for adapter dimers and fragment distribution on a Bioanalyzer HS chip (Agilent Technologies, USA). Each sample was sequenced on an Illumina HiSeq 2500 generating approx. 380–400 M 100 bp paired-end reads. Raw read data were adapter trimmed (Trim Galore!), mapped using bwa [40] to the human reference genome (hs37d5) followed by removal of duplicate reads. Methylation levels of CpGs and GpCs, respectively, were called using Bis-SNP [44]. Cytosines in a GCG context were excluded from further analysis. Bisulfite conversion efficiency was checked genome wide with all cytosines in an HCH context.

NOMe-seq peaks were called with in-house scripts (to be published, https://github.com/karl616/gNOMePeaks/releases/tag/v0.1-alpha): After segmenting the genome with a two-state binomial HMM into putative NDRs and background regions, the former were contrasted to flanking background regions with Fisher’s exact test. Finally, p values were adjusted by calculating empirical false-discovery rates (eFDR) by contrasting to NDRs based on shuffled GCH methylation values. Only NDRs with eFDR below 0.01 were kept.

### Targeted deep bisulfite sequencing and targeted deep NOMe-seq

For regular bisulfite sequencing (BS-seq), 500 ng of DNA was bisulfite-converted using the EZ DNA Methylation-Gold Kit (Zymo Research) according to the manufacturer’s instructions. TrueMethyl Seq kit (CEGX) was used for discriminating 5mC and 5hmC following the manufacturer’s protocol except that 300 ng genomic DNA was used as input for purification and denaturation steps, and split equally before DNA oxidation into oxBS and BS samples. In BS-seq, both 5mC and 5hmC are read as cytosine, while in oxBS-seq, only 5mC is read as cytosine, and thus, 5hmC is inferred from BS- and oxBS-seq comparison. Locus-specific bisulfite amplicon libraries were amplified by PCR employing bisulfite-tagged primers designed using the MethPrimer [45] and BiSearch [46, 47] tools (see primer and PCR details below) and HotStarTaq Master Mix (Qiagen). A second PCR was performed to add sample-specific barcode sequences (MID, multiplex identifiers) and universal linker tags (454 adaptor sequences). Samples were prepared and sequenced on a Roche/454 GS Junior system (Roche Diagnostics) as described elsewhere [48], and special filter settings were applied to increase the yield of reads [49]. For BS- and oxBS-Seq, automated CpG methylation analysis was performed using the Amplikyzer software [50] with minimum bisulfite conversion rate set to 95 %, leading to an average of 2192 reads per sample (minimum 576). For targeted NOMe-seq, both CpG-endogenous and GpC-enzymatic methylation were analyzed with Amplikyzer software [50], obtaining an average of 1409 reads per sample (minimum 176). To assess NOMe-seq efficiency and specificity, sequencing of reference regions within *HSPA5* (mainly open chromatin) and *MLH1* (transition of open to closed chromatin) were performed. Methylated fraction is the percentage of methylated molecules at a specific CpG site as represented by reads in deep bisulfite sequencing. The number of replicates and tested loci are summarized in Additional file 11: Table S5. Primers and PCR conditions for targeted bisulfite sequencing and NOMe-seq can be found in Additional file 12: Table S6.

### Histone modification ChIP-seq

Formaldehyde-fixed cell pellets were resuspended in 1 ml of Farnham laboratory buffer (5 mM PIPES, pH 8; 85 mM KCl; 0.5 % Igepal CA-630) supplemented with protease inhibitors. Nuclei were isolated using the NEXSON approach [51]. The nuclei pellet was resuspended in 1 ml of shearing buffer (10 mM Tris–HCl, pH 8; 0.1 % SDS; 1 mM EDTA) supplemented with protease inhibitor cocktail, and chromatin was sheared using a focused ultrasonicator (Covaris S220). ChIP was performed using an automated liquid handler (SX-8G Compact IP-Star, Diagenode) and the Auto Histone ChIP-seq kit (Diagenode, C01010022) with the following parameters: ChIP indirect method, 200 µl volume of ChIP reactions, 13-h antibody incubation, 4 h beads incubation, 5-min washes (all steps at 4 °C). Eluates were decrosslinked, and DNA was purified using MinElute columns (Qiagen, 28006). The following antibodies were used for ChIP-seq (1 μg per ChIP reaction): anti-H3K27ac (C15410196), anti-H3K4me3 (C15410003), anti-H3K4me1 (C15410194), anti-H3K36me3 (C15410192), anti-H3K9me3 (C15410193) and anti-H3K27me3 (C15410195), all from Diagenode. Sequencing libraries were prepared using the NEBNext Ultra DNA Library Prep kit for Illumina (E7370S, NEB) following the manufacturer’s instructions. Size selection after adapter ligation was omitted, and DNA was amplified by 10 PCR cycles. Final libraries were sequenced on an Illumina HiSeq 2500 generating 50-bp paired-end reads.

Raw reads were aligned with bwa [40] to the human reference genome (hs37d5). Coverage tracks of ChIP-seq samples were calculated as the number of sequenced fragments per 25 bp bin, using the tool bamCoverage from the deepTools suite [52]. Coverage was normalized to 1 × sequencing depth with an effective genome size of 2.9e9. log2 ratio tracks of ChIP over input chromatin signal were computed as log2 ratio of the number of fragments per 25 bp bin, normalized by total read counts per sample, using bamCompare from deepTools. The ChIP over input log2 ratios at DMRs ± 1 kb flanking regions were then visualized as heatmap with the tools computeMatrix and heatmapper from the deepTools suite. All DMRs were scaled to a length of 1 kb. DMRs plus flanks were clustered by bin scores across all six histone modifications and two biological replicates per cell type using the *k*-means algorithm (*k* = 3).

### Data deposition and visualization

Genome raw data from human subjects have been deposited at the European Genome-phenome Archive (EGA, http://www.ebi.ac.uk/ega/), which is hosted at the EBI (Study Accession ID: EGAS00001001595, Dataset Accession ID: EGAD00001002201). To receive access to these controlled data, applications can be addressed to the DEEP Data Access Committee (http://www.deutsches-epigenom-programm.de/data-access/).

### GREAT analysis of enriched genomic annotations

The bioinformatic tool GREAT (Genomic Regions Enrichment of Annotations Tool) was used to investigate the function of differentially methylated regions by analyzing the annotations of the nearby genes [15]. GREAT uses a two-step process. “First, every gene is assigned a regulatory domain of a minimum distance upstream and downstream of the transcription start site (regardless of other nearby genes). The gene regulatory domain is extended in both directions to the nearest gene’s basal domain but no more than the maximum extension in one direction” (great.stanford.edu). We used the default settings for GREAT’s basal plus extension with limits set to 5 kb upstream, 1 kb downstream and an additional distant limit of 1000 kb. “Then, each genomic region is associated with all genes whose regulatory domain it overlaps” (great.stanford.edu). Data on topologically associated domains were taken from [16].

### Regulatory gene network analysis

The interaction network was computed using GeneMANIA software [53] using 245 network data sources: the default networks in the categories genetic interactions (7/7), pathway (6/6), physical interactions (190/190) and predicted (41/42), plus the network consolidated pathways-2013 under the attributes category to allow gene-set enrichment analysis. The network was generated allowing the return of a maximum of 10 attributes and 0 related genes.

### Western blotting

Nuclear extracts from samples were prepared using gentle lysis with 0.1 % NP-40 for 5 min. Protein concentrations of the nuclear extracts were determined by Optiblot Bradford protein assay (Abcam), and 15 μg of extracts was subjected to SDS–polyacrylamide gel electrophoresis on 4–12 % Bis–Tris polyacrylamide gels with MOPS running buffer (Invitrogen). Proteins were transferred onto PVDF membranes (BioRad), which were then blocked by incubation for 1 h with 5 % nonfat dry milk in PBS with 0.1 % Tween 20 (PBS-T). Primary antibodies (TET2 mAb clone 21F11, Active Motif 61389; TET3 rabbit polyclonal IgG, Thermo-Scientific, PA5-31960) were diluted 1:1000 in 1 % BSA in PBS-T and incubated overnight at 4 °C. The membranes were washed with PBS-T, incubated for 1 h at room temperature with anti-rabbit or anti-mouse-HRP-IgG (Jackson ImmunoResearch), washed in PBS-T, and antibody binding was detected by chemiluminescence ECL-Pus blot detection system (GE-Healthcare).

### TET activity assay

TET functional enzyme activity was quantified in triplicate using the commercially available TET Hydroxylase Activity Quantification Kit (Abcam) from nuclear extracts according to manufacturer’s instructions. Fluorescence readings were taken on a FluoStar Galaxy microplate reader (BMG).

The competitive inhibitor of TET activity (2S)-Octyl-α-hydroxyglutarate was purchased from Cayman Chemical. (2S)-Octyl-α-hydroxyglutarate is a cell-permeable derivative of the L-isomer of 2-HG that has been described to be the primarily active isomer in the inhibition of TET enzymes [24].

### Flow cytometry

Antibodies given in Additional file 13: Table S7 were used for flow cytometry of surface antigens. The analysis was performed on a Becton–Dickinson FACS Canto II flow cytometer according to standard protocols. To specifically stain apoptotic and necrotic cells, the ANNEXIN V-FITC/7-AAD kit from Becton–Dickinson was used according to manufacturer’s instructions.

## Additional files

10.1186/s13072-016-0079-z Scatter plot of the mean log2 fold changes obtained by BLUEPRINT for three pairs of monocytes/macrophages vs. the mean log2 fold changes of the two pairs of monocytes/macrophages analyzed by DEEP (r = 0.68).
10.1186/s13072-016-0079-z List of statistically significantly overrepresented GO terms (GO biological process data set) for genes up-regulated during ​monocyte-to-macrophage differentiation (log2Fc > 2) in the Blueprint study (Saeed et al. 2014) but with unchanged expression in the present study (log2Fc < 2). Terms with fold enrichment >2 are shown. In the reverse analysis (genes up-regulated in the present study, but unchanged in the Blueprint study), no significant enrichment of GO terms was found.
10.1186/s13072-016-0079-z Regulation of miRNAs during differentiation.
10.1186/s13072-016-0079-z Correlation plot of gene expression changes and DNA methylation changes around the transcription start site (± 1 kb). Each dot represents a gene. The x-coordinate shows the mean change of DNA methylation during monocyte-to-macrophage differentiation in the regions ± 1 kb around the TSS of the corresponding genes. The y-coordinate represents the log2 fold change in its expression.
10.1186/s13072-016-0079-z Overview over all identified DMRs and their characteristics.
10.1186/s13072-016-0079-z Validation of DMRs by deep bisulfite sequencing. Comparative methylation plots of monocytes and macrophage from 4 independent donor samples (Hm03, Hm04, Hm05 and Hm06). Samples sorted by overall methylation. DMR33 is shown in the main text. Related to Fig. 2d and 2e.
10.1186/s13072-016-0079-z Distance of DMRs to the transcription start sites (TSS) of their associated genes (GREAT).
10.1186/s13072-016-0079-z (Tab 1) Gene Ontology analysis of genes that are associated with DMRs. (Tab 2) DMR associated genes that belong to an identified GO category and that were used for the GREAT analysis.
10.1186/s13072-016-0079-z Time course of DNA demethylation. Comparative methylation plots obtained by targeted deep bisulfite sequencing of 10 DMRs show a rapid decline in DNA methylation during monocyte-to-macrophage differentiation (time points: 0 h, 12 h, 24 h, 36 h, 48 h, 60 h and 72 h). Samples sorted by differentiation time.
10.1186/s13072-016-0079-z Time course of chromatin accessibility (GpC methylation) and DNA CpG methylation in 4 DMRs during monocyte-to-macrophage differentiation. The transition from lower to higher GpC methylated fraction is indicative of an increase in chromatin accessibility. Average CpG and GpC methylated fractions in Hm10 donor monocytes (0 h) and cells collected at different time points during differentiation into macrophages (6 h, 12 h, 18 h, 36 h, 48 h and 72 h). GCG motifs were excluded due to ambiguity between CpG- endogenous- and GpC-enzymatic-methylation. Related to Fig. 5b.
10.1186/s13072-016-0079-z Overview over the donors and sample nomenclature used in the analyses presented in the manuscript.
10.1186/s13072-016-0079-z Primer sequences and PCR conditions for targeted bisulfite sequencing and NOMe-Seq analysis.
10.1186/s13072-016-0079-z Antibodies used for flow cytometry of surface antigens.
10.1186/s13072-016-0079-z Time course of 5hmC (5-hydroxymethylcytosine) and 5mC (5-methylcytosine) in 2 DMRs during monocyte-to-macrophage differentiation. Average cytosine variant levels in Hm06 (A and B) and Hm10 (C) donor monocytes (0 h) and cells collected at different time points during differentiation into macrophages. (A) DMR33, (B and C) DMR13. Related to Fig. 5c.

## Abbreviations

2-HG: 2-hydroxyglutaric acid
ChIP: chromatin immunoprecipitation
CSF1: colony-stimulating factor 1
CSF1R: CSF1 receptor
DMR: differentially methylated region
ENCODE: Encyclopedia of DNA Elements
eRNA: enhancer RNA
FANTOM: Functional ANnoTation Of the Mammalian genome
γ-IFN: interferon gamma
GO: gene ontology
GREAT: Genomic Regions Enrichment of Annotations
H3K27ac: histone H3 lysine 27 acetylation
H3K27me3: histone H3 lysine 27 tri-methylation
H3K36me3: histone H3 lysine 36 tri-methylation
H3K4me1: histone H3 lysine 4 mono-methylation
H3K4me3: histone H3 lysine 4 tri-methylation
H3K9me3: histone H3 lysine 9 tri-methylation
IHEC: International Human Epigenome Consortium
log2FC: log2 fold change
LPS: lipopolysaccharides
NOMe: nucleosome occupancy and methylation
RISC: RNA-induced silencing complex
SRF: serum response factor
TAD: topologically associating domain
TET: ten eleven translocation
TF: transcription factor
TNF: tumor necrosis factor
WGBS: whole-genome bisulfite sequencing
