# Supplementary figures and images for: Epigenetic dynamics of monocyte-to-macrophage differentiation
Source: Epigenetics Chromatin. 2016 Jul 29;9:33. doi: 10.1186/s13072-016-0079-z (PMC4967341; doi:10.1186/s13072-016-0079-z)

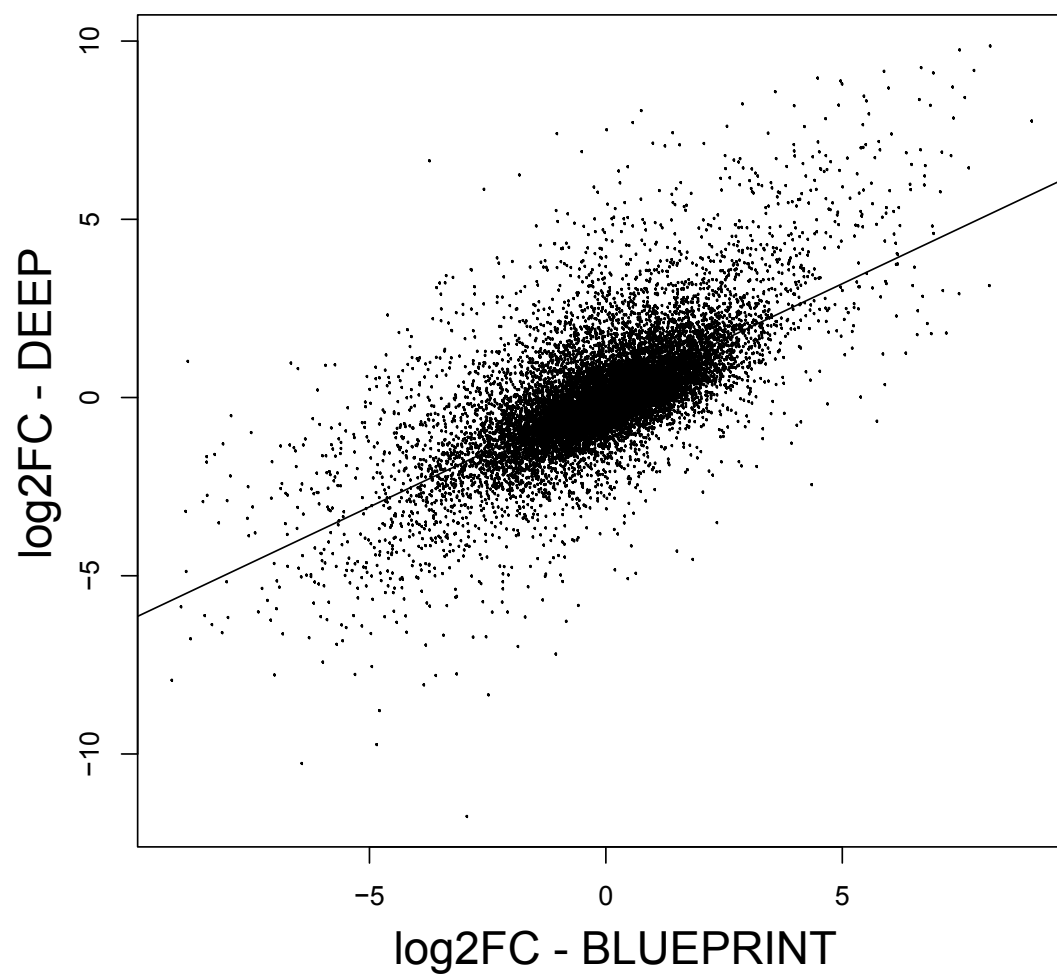

Supplement: Supplementary file 1 — 10.1186/s13072-016-0079-z Scatter plot of the mean log2 fold changes obtained by BLUEPRINT for three pairs of monocytes/macrophages vs. the mean log2 fold changes of the two pairs of monocytes/macrophages analyzed by DEEP (r = 0.68). [file 13072_2016_79_MOESM1_ESM.pdf]

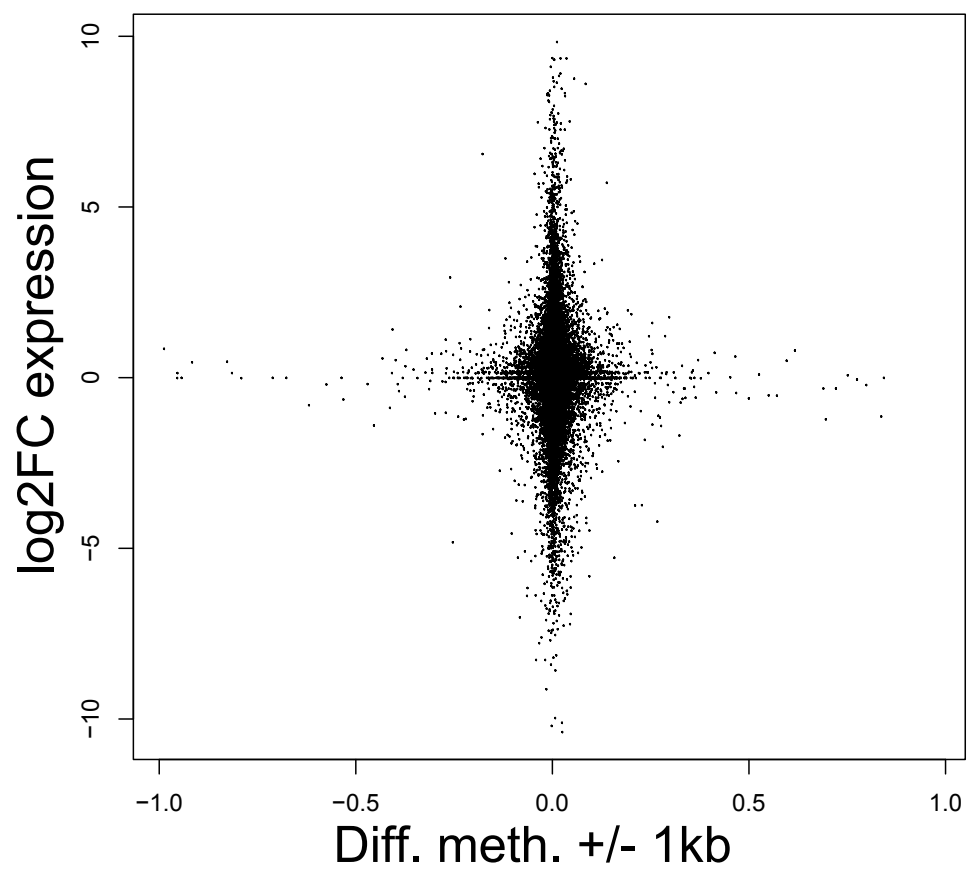

Supplement: Supplementary file 4 — 10.1186/s13072-016-0079-z Correlation plot of gene expression changes and DNA methylation changes around the transcription start site (± 1 kb). Each dot represents a gene. The x-coordinate shows the mean change of DNA methylation during monocyte-to-macrophage differentiation in the regions ± 1 kb around the TSS of the corresponding genes. The y-coordinate represents the log2 fold change in its expression. [file 13072_2016_79_MOESM4_ESM.pdf]

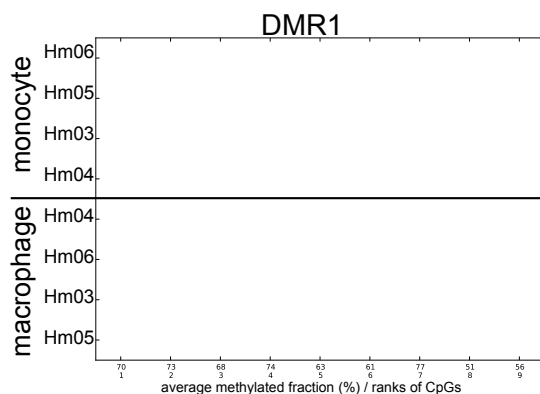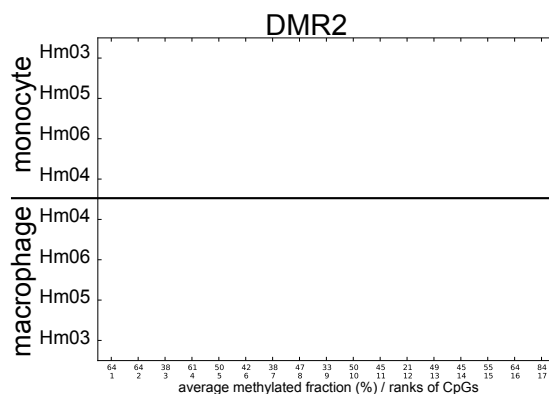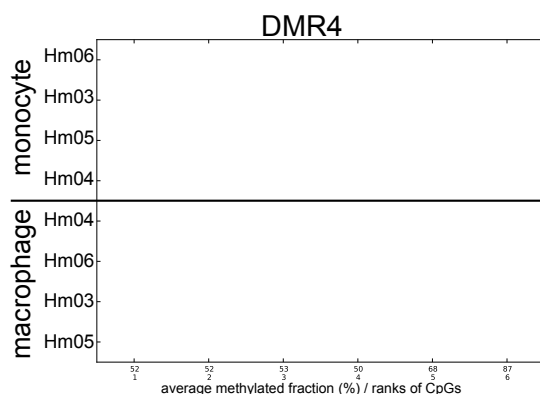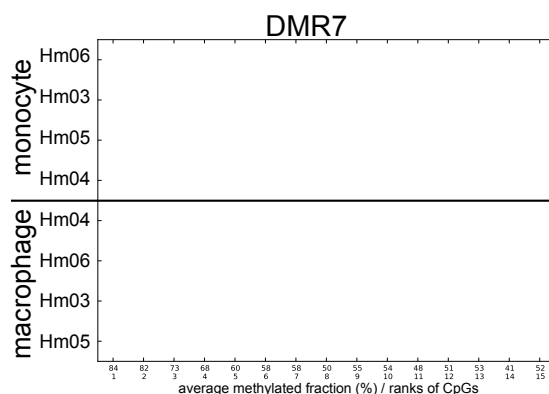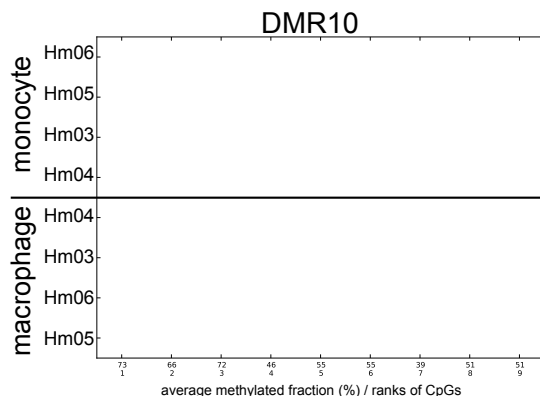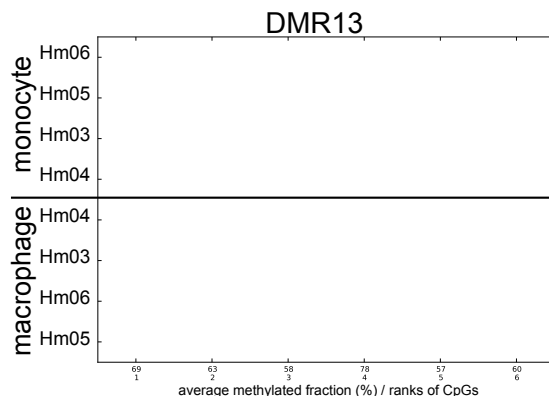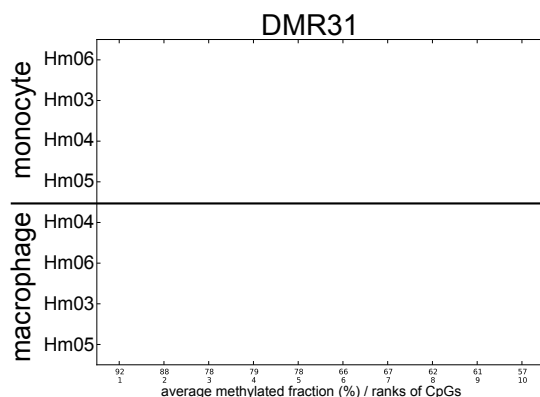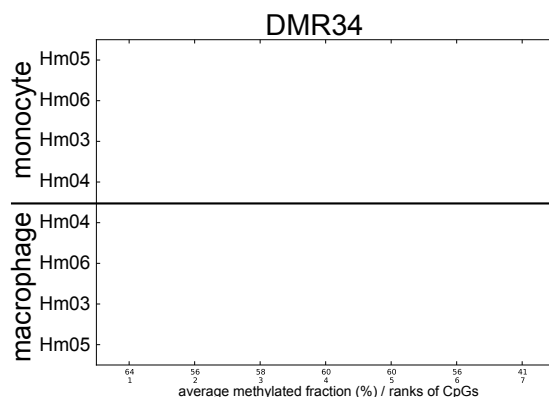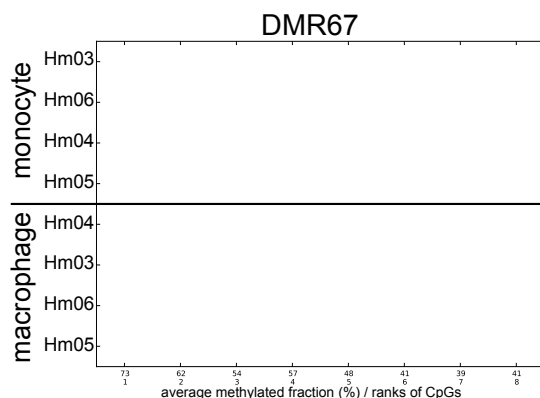

Supplement: Supplementary file 6 — 10.1186/s13072-016-0079-z Validation of DMRs by deep bisulfite sequencing. Comparative methylation plots of monocytes and macrophage from 4 independent donor samples (Hm03, Hm04, Hm05 and Hm06). Samples sorted by overall methylation. DMR33 is shown in the main text. Related to Fig. 2d and 2e. [file 13072_2016_79_MOESM6_ESM.pdf]

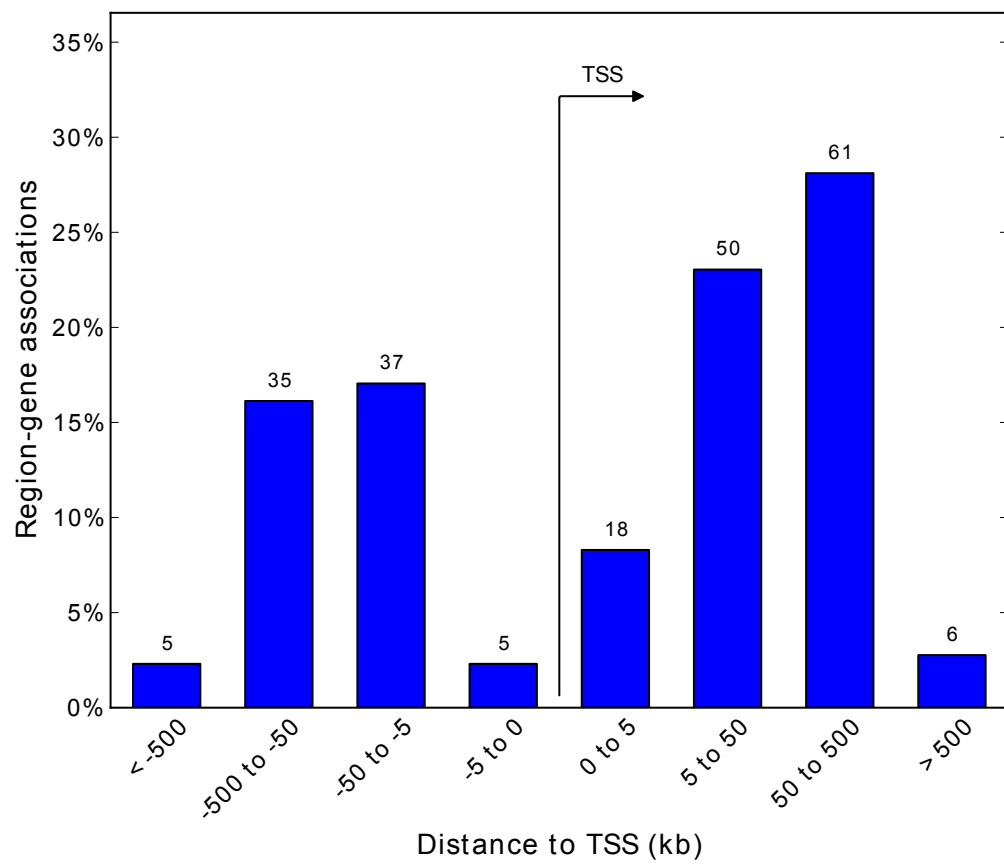

Supplement: Supplementary file 7 — 10.1186/s13072-016-0079-z Distance of DMRs to the transcription start sites (TSS) of their associated genes (GREAT). [file 13072_2016_79_MOESM7_ESM.pdf]

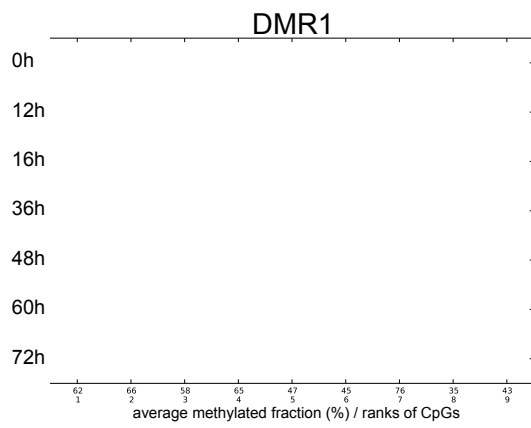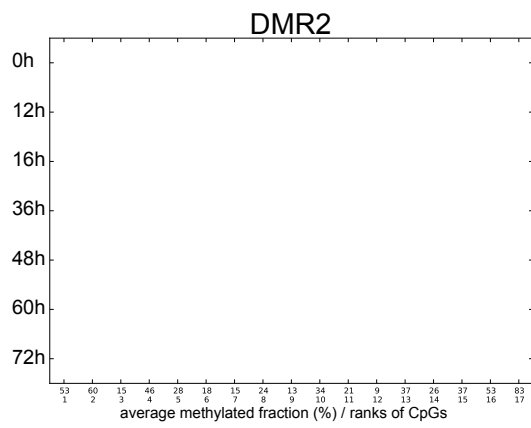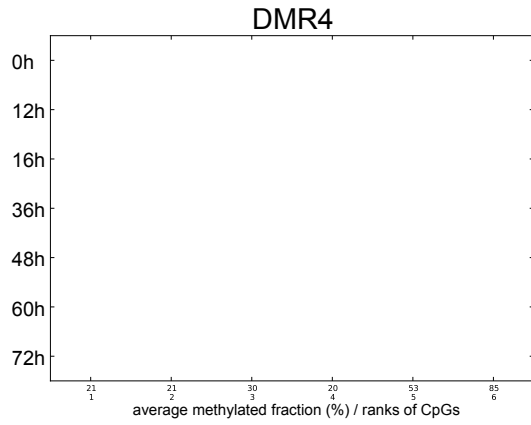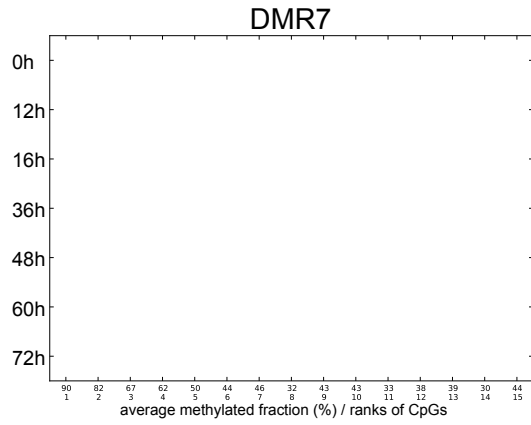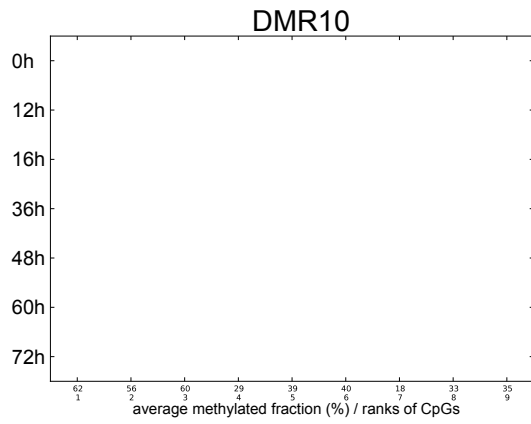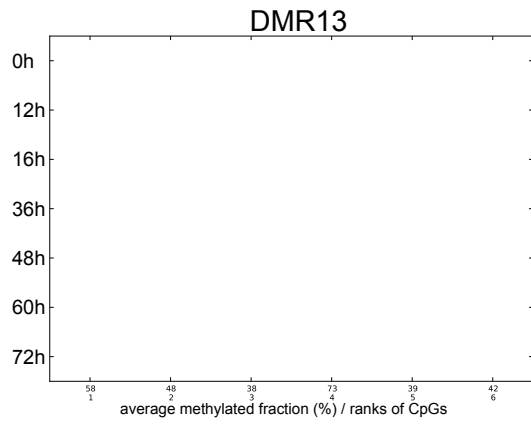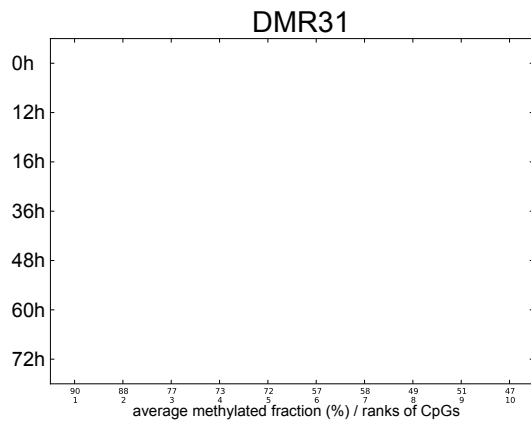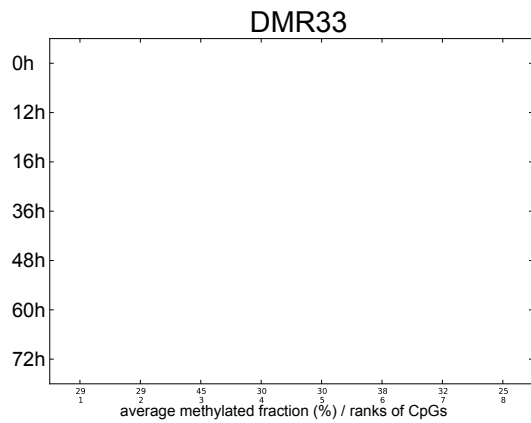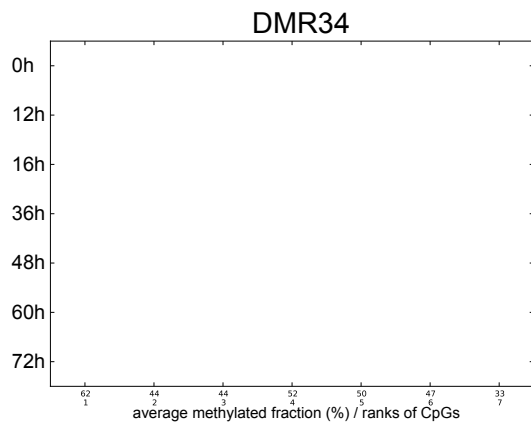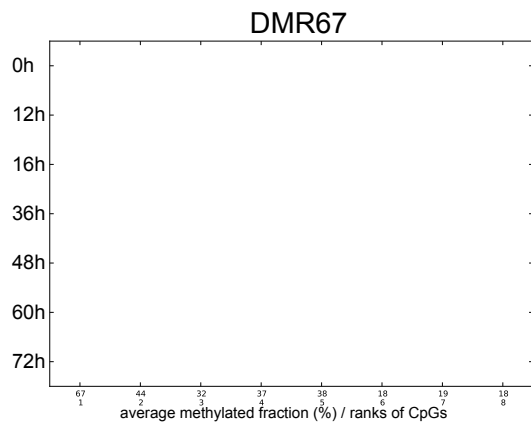

Supplement: Supplementary file 9 — 10.1186/s13072-016-0079-z Time course of DNA demethylation. Comparative methylation plots obtained by targeted deep bisulfite sequencing of 10 DMRs show a rapid decline in DNA methylation during monocyte-to-macrophage differentiation (time points: 0 h, 12 h, 24 h, 36 h, 48 h, 60 h and 72 h). Samples sorted by differentiation time. [file 13072_2016_79_MOESM9_ESM.pdf]

**DMR1**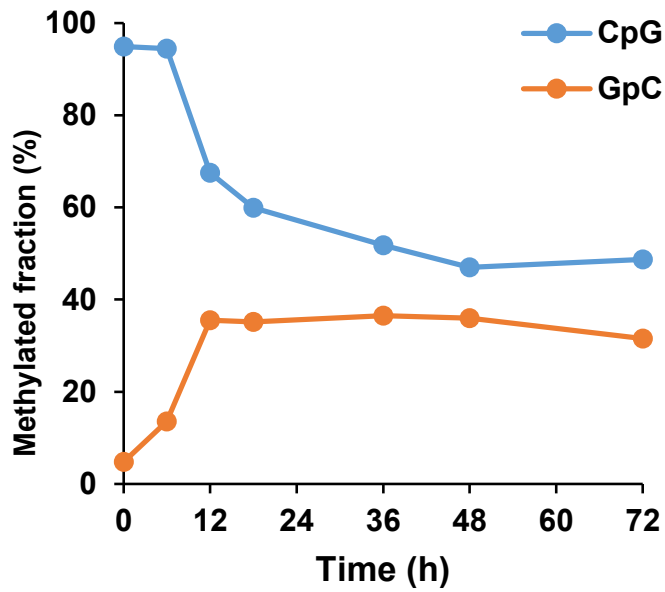**DMR2**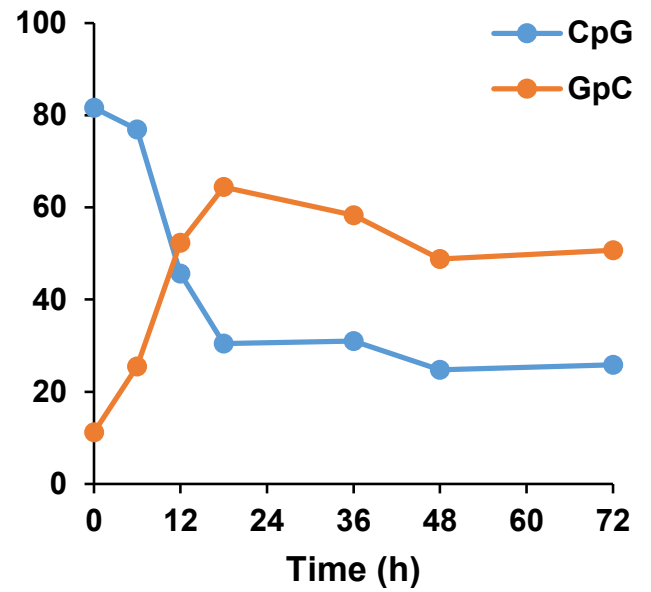**DMR10**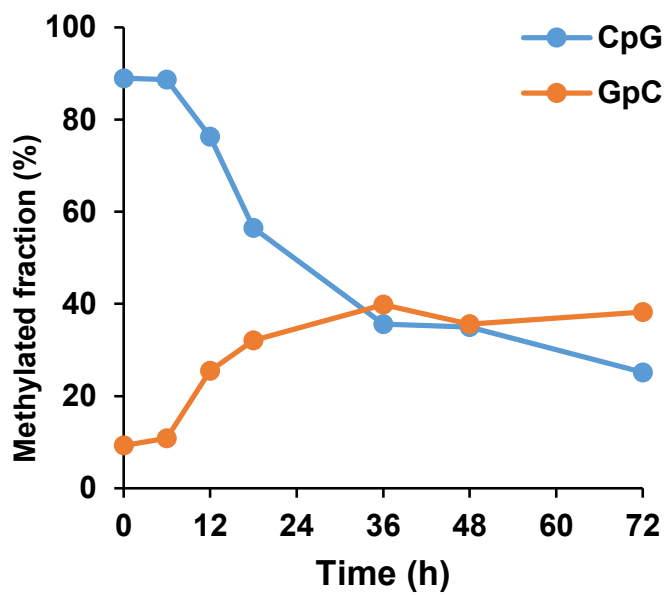**DMR13**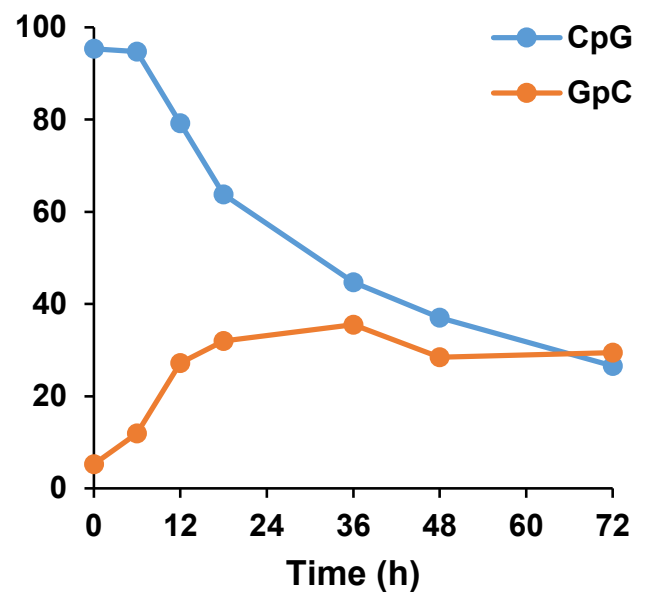

Supplement: Supplementary file 10 — 10.1186/s13072-016-0079-z Time course of chromatin accessibility (GpC methylation) and DNA CpG methylation in 4 DMRs during monocyte-to-macrophage differentiation. The transition from lower to higher GpC methylated fraction is indicative of an increase in chromatin accessibility. Average CpG and GpC methylated fractions in Hm10 donor monocytes (0 h) and cells collected at different time points during differentiation into macrophages (6 h, 12 h, 18 h, 36 h, 48 h and 72 h). GCG motifs were excluded due to ambiguity between CpG- endogenous- and GpC-enzymatic-methylation. Related to Fig. 5b. [file 13072_2016_79_MOESM10_ESM.pdf]

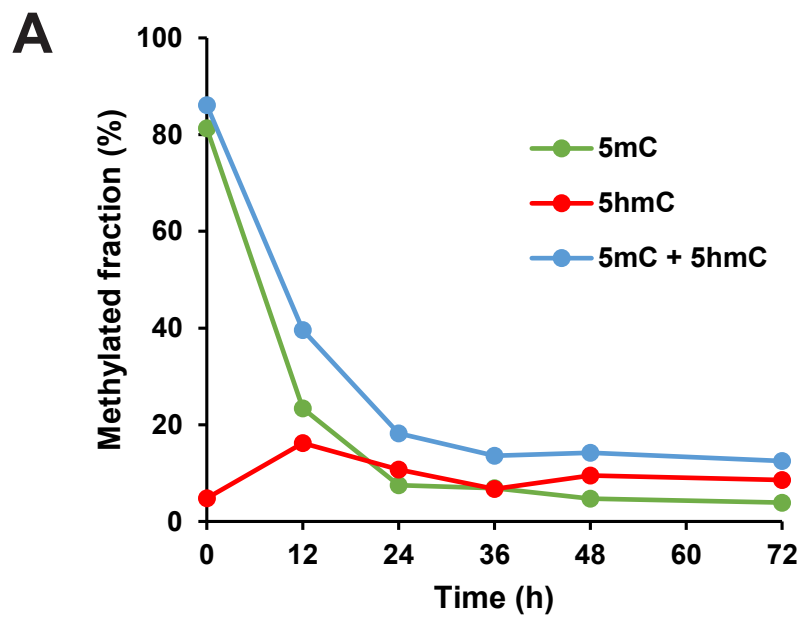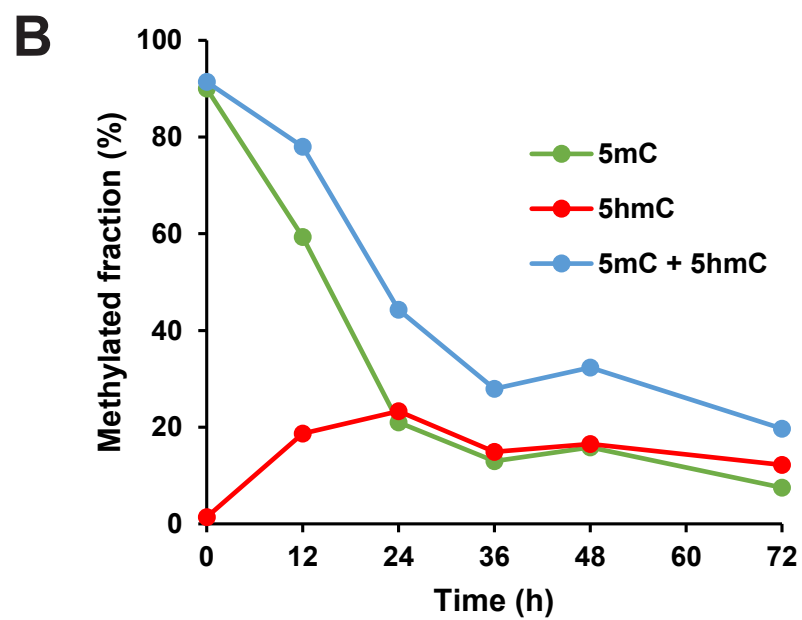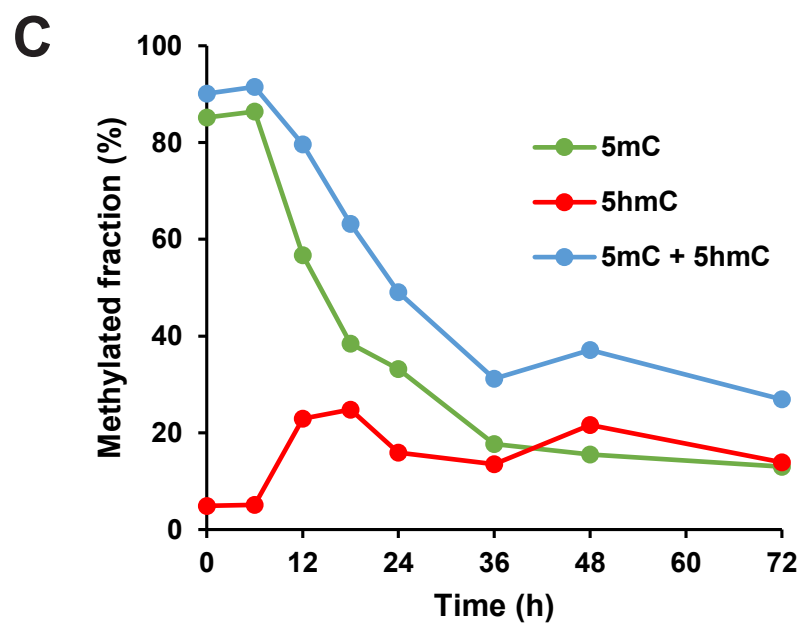

Supplement: Supplementary file 14 — 10.1186/s13072-016-0079-z Time course of 5hmC (5-hydroxymethylcytosine) and 5mC (5-methylcytosine) in 2 DMRs during monocyte-to-macrophage differentiation. Average cytosine variant levels in Hm06 (A and B) and Hm10 (C) donor monocytes (0 h) and cells collected at different time points during differentiation into macrophages. (A) DMR33, (B and C) DMR13. Related to Fig. 5c. [file 13072_2016_79_MOESM14_ESM.pdf]
