# Supplementary material for: Epigenetic dynamics of monocyte-to-macrophage differentiation
Source: Epigenetics Chromatin. 2016 Jul 29;9:33. doi: 10.1186/s13072-016-0079-z (PMC4967341; doi:10.1186/s13072-016-0079-z)
Supplement: Supplementary file 2 — 10.1186/s13072-016-0079-z List of statistically significantly overrepresented GO terms (GO biological process data set) for genes up-regulated during ​monocyte-to-macrophage differentiation (log2Fc > 2) in the Blueprint study (Saeed et al. 2014) but with unchanged expression in the present study (log2Fc < 2). Terms with fold enrichment >2 are shown. In the reverse analysis (genes up-regulated in the present study, but unchanged in the Blueprint study), no significant enrichment of GO terms was found. [file 13072_2016_79_MOESM2_ESM.docx]

| **GO Biological Process** | **# genes** | **Expected** | **Fold Enrichment** | ***p* value** |
| --- | --- | --- | --- | --- |
| **fatty acid beta-oxidation** | 10 | 1,43 | > 5 | 1,97E-02 |
| **steroid biosynthetic process** | 14 | 2,94 | 4,77 | 1,87E-02 |
| **organic acid catabolic process** | 22 | 5,25 | 4,19 | 2,54E-04 |
| **carboxylic acid catabolic process** | 22 | 5,25 | 4,19 | 2,54E-04 |
| **small molecule catabolic process** | 26 | 6,97 | 3,73 | 1,45E-04 |
| **small molecule biosynthetic process** | 31 | 10,5 | 2,95 | 1,18E-03 |
| **carboxylic acid metabolic process** | 54 | 22,3 | 2,42 | 2,82E-05 |
| **oxoacid metabolic process** | 60 | 25,34 | 2,37 | 8,19E-06 |
| **organic acid metabolic process** | 60 | 25,81 | 2,32 | 1,60E-05 |
| **oxidation-reduction process** | 62 | 26,88 | 2,31 | 1,13E-05 |
| **carbohydrate metabolic process** | 45 | 20,64 | 2,18 | 1,03E-02 |
| **small molecule metabolic process** | 116 | 58,04 | 2 | 2,95E-09 |
|  |  |  |  |  |
| **Table S1:** List of statistically significantly overrepresented GO terms (GO biological process data set) for genes up-regulated during monocyte-to-macrophage differentiation (log2Fc>2) in the Blueprint study (Saeed et al. 2014) but with unchanged expression in the present study (log2Fc<2). Terms with fold enrichment >2 are shown. In the reverse analysis (genes upregulated in the present study, but unchanged in the Blueprint study) showed no significant enrichment of GO terms. | | | | |
