# Supplementary material for: Epigenetic dynamics of monocyte-to-macrophage differentiation
Source: Epigenetics Chromatin. 2016 Jul 29;9:33. doi: 10.1186/s13072-016-0079-z (PMC4967341; doi:10.1186/s13072-016-0079-z)
Supplement: Supplementary file 3 — 10.1186/s13072-016-0079-z Regulation of miRNAs during differentiation. [file 13072_2016_79_MOESM3_ESM.docx]

| miRNA | base mean | log2FC | adjusted p-value |
| --- | --- | --- | --- |
| hsa-miR-99b-5p | 27436 | 6.61 | 4.26E-62 |
| hsa-miR-34a-5p | 3833 | 8.56 | 6.32E-53 |
| hsa-miR-125a-5p | 21750 | 5.73 | 4.20E-33 |
| hsa-miR-221-5p | 2250 | 4.16 | 2.79E-13 |
| hsa-miR-99b-3p | 152 | 6.23 | 6.38E-11 |
| hsa-miR-150-5p | 400 | -4.37 | 1.45E-10 |
| hsa-miR-221-3p | 21820 | 2.67 | 1.12E-09 |
| hsa-miR-211-5p | 228 | 7.49 | 1.92E-09 |
| hsa-miR-222-3p | 16459 | 2.99 | 4.47E-08 |
| hsa-miR-1286 | 78 | 7.55 | 2.55E-07 |
| hsa-miR-451a | 83 | -5.35 | 5.05E-07 |
| hsa-miR-511-5p | 155 | 5.80 | 6.65E-07 |
| hsa-let-7e-3p | 58 | 5.62 | 1.40E-06 |
| hsa-miR-132-3p | 955 | 3.32 | 2.27E-06 |
| hsa-miR-146a-5p | 241151 | 3.66 | 1.50E-05 |
| hsa-miR-146b-3p | 896 | 2.96 | 2.91E-05 |
| hsa-miR-34a-3p | 35 | 6.64 | 2.91E-05 |
| hsa-miR-212-5p | 171 | 3.57 | 3.70E-05 |
| hsa-miR-147b | 81 | 4.46 | 4.32E-05 |
| hsa-miR-146b-5p | 477329 | 3.83 | 6.13E-05 |
| hsa-miR-22-3p | 148401 | 2.16 | 6.58E-05 |
| hsa-miR-212-3p | 130 | 3.65 | 1.44E-04 |
| hsa-miR-1910-5p | 56 | -4.35 | 2.17E-04 |
| hsa-miR-409-3p | 22 | -5.26 | 9.43E-04 |
| hsa-miR-486-5p | 52917 | -3.14 | 1.10E-03 |
| hsa-miR-3065-3p | 41 | 3.79 | 1.38E-03 |
| hsa-miR-23a-5p | 1171 | -2.98 | 1.68E-03 |
| hsa-miR-511-3p | 21 | 5.08 | 1.71E-03 |
| hsa-miR-30a-5p | 1366 | 3.21 | 2.05E-03 |
| hsa-miR-4485-3p | 54199 | -4.67 | 4.79E-03 |
| hsa-miR-223-5p | 1239 | -2.82 | 5.48E-03 |
| hsa-miR-133a-3p | 147 | 3.41 | 6.18E-03 |
| hsa-miR-449c-5p | 42 | -4.00 | 8.19E-03 |
| hsa-miR-2116-3p | 74 | 3.11 | 8.28E-03 |
| hsa-miR-1-3p | 28 | 4.63 | 1.39E-02 |
| hsa-let-7e-5p | 17170 | 2.82 | 1.53E-02 |
| hsa-miR-1302 | 12 | 5.08 | 1.66E-02 |
| hsa-miR-365a-3p | 816 | 2.26 | 1.66E-02 |
| hsa-miR-365b-3p | 816 | 2.26 | 1.66E-02 |
| hsa-miR-210-3p | 64 | 2.85 | 1.92E-02 |
| hsa-miR-130a-3p | 345 | -2.32 | 3.30E-02 |
| hsa-miR-6503-3p | 381 | -2.25 | 3.70E-02 |
| hsa-miR-342-3p | 16591 | 2.17 | 3.93E-02 |
| hsa-miR-486-3p | 159 | -2.76 | 4.83E-02 |
| hsa-miR-210-5p | 8 | 4.65 | 4.89E-02 |
| hsa-miR-4787-3p | 21 | 3.84 | 4.89E-02 |

**Table S2:** List of significantly regulated miRNAs during monocyte to macrophage differentiation (p<0.05).
