# Supplementary material for: Epigenetic dynamics of monocyte-to-macrophage differentiation
Source: Epigenetics Chromatin. 2016 Jul 29;9:33. doi: 10.1186/s13072-016-0079-z (PMC4967341; doi:10.1186/s13072-016-0079-z)
Supplement: Supplementary file 12 — 10.1186/s13072-016-0079-z Primer sequences and PCR conditions for targeted bisulfite sequencing and NOMe-Seq analysis. [file 13072_2016_79_MOESM12_ESM.docx]

| DMR | PCR target | Sequence (5'-3') | Ta (°C) | 2^nd^ PCR |
| --- | --- | --- | --- | --- |
| 1 | chr15:68,605,231- | BS-F: cttgcttcctggcacgagAGTTTAGTTAGGGTTGGTTGG | 53 | 3-step |
|  | 68,605,519 | BS-R: caggaaacagctatgacAAAAAAACACAATAACATAATCACTC |  |  |
|  |  | NOMe-F: cttgcttcctggcacgagAGYTTAGTTAGGGYTGGYTGG |  |  |
|  |  | NOMe-R: caggaaacagctatgacAAAAAARCACAATARCATAATCACTC |  |  |
| 2 | chr8:141,598,888- | BS-F: cttgcttcctggcacgagGGTTTTTTGTATTTAAGGTTATATTTATTT | 58 | 2-step |
|  | 141,599,264 | BS-R: caggaaacagctatgacATAACACAAAATCTACTCCACATCC |  |  |
|  |  | NOMe-F: cttgcttcctggcacgagGGYTTTTTGYATTTAAGGYTATATTTATTT |  |  |
|  |  | NOMe-R: caggaaacagctatgacATARCACAAAATCTRCTCCACATCC |  |  |
| 4 | chr2:230,272,098- | BS-F: cttgcttcctggcacgagGGATTTTTTGGTTTTTATGGTTTTT | 59 | 2-step |
|  | 230,272,349 | BS-R: caggaaacagctatgacACTTTTAACTTTCTATTCCCCAAACTT |  |  |
| 7 | chr10:63,808,803- | BS-F: cttgcttcctggcacgagATAAAGTTTTTTAAATAAAATAAATAGTTT | 48 | 2-step |
|  | 63,809,196 | BS-R: caggaaacagctatgacAAAACTACAACATATAACTACTCCC |  |  |
| 10 | chr10:80,732,398- | BS-F: cttgcttcctggcacgagTTTGTTTTGGTTATATTAGTTTTTT | 53 | 2-step |
|  | 80,732,747 | BS-R: caggaaacagctatgacCTCCCCTTTAATAAAACCCCA |  |  |
|  |  | NOMe-F: cttgcttcctggcacgagTTTGYTTTGGTTATATTAGYTTTTT |  |  |
|  |  | NOMe-R: caggaaacagctatgacCTCCCCTTTAATAAARCCCCA |  |  |
| 13 | chr7:73,512,680- | BS-F: cttgcttcctggcacgagATTGATTAAGTTGGATTAGAGGATA | 54 | 2-step |
|  | 73,512,882 | BS-R: caggaaacagctatgacTTTCACTCTTCCTATAAAACTAAAAA |  |  |
|  |  | NOMe-F: cttgcttcctggcacgagATTGATTAAGYTGGATTAGAGGATA |  |  |
|  |  | NOMe-R: caggaaacagctatgacTTTCACTCTTCCTATAAAACTAAAAR |  |  |
| 31 | chr2:9,556,024- | BS-F: cttgcttcctggcacgagGGTATATGTGGTATATGGGAGGTT | 52 | 3-step |
|  | 9,556,369 | BS-R: caggaaacagctatgacAAAAAACAAATAATAAATAAAACCTTAAAA |  |  |
| 33 | chr19:13,962,352- | BS-F: cttgcttcctggcacgagTTATTATAGGAAGTTGGGTGTTTTT | 56 | 3-step |
|  | 13,962,525 | BS-R: caggaaacagctatgacCAAATTTCCTTCTCAACCTAACT |  |  |
|  |  | NOMe-F: cttgcttcctggcacgagTTATTATAGGAAGYTGGGTGTTTTT |  |  |
|  |  | NOMe-R: caggaaacagctatgacCAAATTTCCTTCTCARCCTAACT |  |  |
| 34 | chr20:1,307,982- | BS-F: cttgcttcctggcacgagGAGTTGAAGTGATGGAATTTTTATT | 61 | 2-step |
|  | 1,308,263 | BS-R: caggaaacagctatgacTCTAAACCCTACCCTACCTAAACCTA |  |  |
| 67 | chr17:75,319,147- | BS-F: cttgcttcctggcacgagTGGTTTGTTAGTTTTAGGGAAGGT | 55 | 2-step |
|  | 75,319,436 | BS-R: caggaaacagctatgacACTTAATTTATAAAAACCACAAAAC |  |  |

Table S6: Primer sequences and PCR conditions for targeted bisulfite sequencing and NOMe-Seq analysis

BS, bisulfite sequencing; NOMe, nucleosome occupancy and methylome sequencing. F, forward primer; R, reverse primer. Y, C or T; R, A or G. Lower case sequences correspond to tag sequences.

Ta (°C): annealing temperature for the first PCR; 1st PCR conditions: 95°C 15 min, 40× (95°C 30 s, Ta °C 1 min, 72°C 1min), 72°C 10 min

2nd PCR conditions: 2-step (95°C 15 min, 35× [95°C 30 s, 72°C 1min], 72°C 10 min; 3-step (95°C 15 min, 35× [95°C 30 s, 58°C 30 s, 72°C 1min], 72°C 10 min
