# Supplementary material for: Epigenetic dynamics of monocyte-to-macrophage differentiation
Source: Epigenetics Chromatin. 2016 Jul 29;9:33. doi: 10.1186/s13072-016-0079-z (PMC4967341; doi:10.1186/s13072-016-0079-z)
Supplement: Supplementary file 13 — 10.1186/s13072-016-0079-z Antibodies used for flow cytometry of surface antigens. [file 13072_2016_79_MOESM13_ESM.docx]

| antigen | dye | manufacturer | order # |
| --- | --- | --- | --- |
| HLA-DR | FITC | Becton Dickinson | 347400 |
| CD68 | FITC | Acris | SM1550F |
| CD195 | FITC | Becton Dickinson | 555992 |
| CD71 | FITC | Becton Dickinson | 333151 |
| CD22 | PE | Becton Dickinson | 337899 |
| CD16 | PE | Beckman Coulter | A07766 |
| CD11b | PE | Becton Dickinson | 333142 |
| CD81 | PE | Becton Dickinson | 555676 |
| CD45 | PerCP | Dako | PR701 |
| CD14 | APC | Becton Dickinson | 345787 |
| CD5 | FITC | Becton Dickinson | 345781 |
| CD5 | PE | Becton Dickinson | 345782 |
| CD5 | APC | Becton Dickinson | 345783 |

Table S7: Antibodies used for flow cytometry of surface antigens.
